# Emergence and fragmentation of the alpha-band driven by neuronal network dynamics

**DOI:** 10.1371/journal.pcbi.1009639

**Published:** 2021-12-06

**Authors:** Lou Zonca, David Holcman

**Affiliations:** 1 Sorbonne University, Pierre et Marie Curie Campus, Paris, France; 2 Group of Applied Mathematics and Computational Biology, IBENS, Ecole Normale Supérieure, PSL University, Paris, France; 3 University Of Cambridge, DAMPT and Churchill College CB30DS, United Kingdom; Oxford, UNITED KINGDOM

## Abstract

Rhythmic neuronal network activity underlies brain oscillations. To investigate how connected neuronal networks contribute to the emergence of the *α*-band and to the regulation of Up and Down states, we study a model based on synaptic short-term depression-facilitation with afterhyperpolarization (AHP). We found that the *α*-band is generated by the network behavior near the attractor of the Up-state. Coupling inhibitory and excitatory networks by reciprocal connections leads to the emergence of a stable *α*-band during the Up states, as reflected in the spectrogram. To better characterize the emergence and stability of thalamocortical oscillations containing *α* and *δ* rhythms during anesthesia, we model the interaction of two excitatory networks with one inhibitory network, showing that this minimal topology underlies the generation of a persistent *α*-band in the neuronal voltage characterized by dominant Up over Down states. Finally, we show that the emergence of the *α*-band appears when external inputs are suppressed, while fragmentation occurs at small synaptic noise or with increasing inhibitory inputs. To conclude, *α*-oscillations could result from the synaptic dynamics of interacting excitatory neuronal networks with and without AHP, a principle that could apply to other rhythms.

## Author summary

Brain oscillations, recorded from electroencephalograms characterize behaviors such as sleep, wakefulness, brain evoked responses, coma or anesthesia. The underlying rhythms for these oscillations are associated at a neuronal population level to fluctuations of the membrane potential between Up (depolarized) and Down (hyperpolarized) states. During anesthesia with propofol, a dominant *α*-band (8–12Hz) can emerge or disappear, but the underlying mechanism remains unclear. Using modeling, we report that the *α*-band appears during Up states in neuronal populations driven by short-term synaptic plasticity and synaptic noise. Moreover, we show that three connected neuronal networks representing the thalamocortical loop reproduce the dynamics of the *α*-band, which emerges following the arrest of excitatory stimulations, but that can disappear by increasing inhibitory inputs. To conclude, short-term plasticity in well connected neuronal networks can explain the emergence and fragmentation of the *α*-band.

## Introduction

Electroencephalogram (EEG) is used to monitor the brain activity in various conditions such as sleep [1, 2], coma [3] or meditation [4] and to reveal and quantify the presence of multiple frequency oscillations [5] over time [6]. This analysis can be used to assess the level of consciousness or depth of unconsciousness of the brain during anesthesia [7, 8]. For example, during general anesthesia under propofol, the *α*-band (8–12Hz) emerges as a dominant oscillation [7, 9]. However, the precise mechanisms underlying the emergence or disappearance of this *α*-band remain unknown. Interestingly, when the level of sedation becomes too high, the EEG shows that the *α*-band can get fragmented and even disappear replaced by burst-suppression, consisting of alternation between periods of high frequency activity and iso-electric suppression where the EEG is almost flat [7]. In general, large doses of hypnotic in prolonged anesthesia in rodents alters brain synaptic architecture [10], confirming the need to avoid over sedation. Burst-suppression is a motif associated with a too deep anesthesia and its presence could indicate possible post anesthetic complications, although it has been attributed to ATP depletion [11]. Recently, it was shown that the loss of the *α*-band announces the appearance of burst-suppressions [8], and could be used as a prediction marker for these suppressions. However, this causality between *α*-band suppression and burst-suppressions remains unexplained.

The *α*-band revealed by the EEG signal is associated with the local Up and Down states activity [12–14], which corresponds to depolarized and hyperpolarized neuronal membrane voltage [15]. Alternation between Up and Down states generates slow wave oscillations present in Non-REM sleep, as reported in slices electrophysiology [16] as well as using modeling approaches [17, 18]. Similarly, the emergence of the *α*-band during anesthesia could result from network interactions, as proposed by models based on the Hodgkin-Huxley formalism [19–21].

Since Up and Down states dynamics reflect neuronal activity at the population level [15, 22], we propose here to investigate the emergence and fragmentation of the *α*-band using a modeling approach based on synaptic short-term plasticity [23, 24], which is often used to obtain estimations for burst or interburst durations [25–27]. These models, based on facilitation and depression have recently been used to evaluate the working memory capacity to remember a sequence of words [28].

Here, we use a mean-field neuronal model that accounts for both synaptic short-term dynamics and afterhyperpolarization (AHP) [29] resulting in a refractory period during which neurons stops firing after a burst. As a result, at a population level, AHP can modify the type of oscillations [30], from waxing and waning spindle oscillations to slow waves.

We first study a single, two and then three interacting neuronal networks, a minimal configuration revealing the coexistence of *α*-oscillations with switching between Up and Down states. As we shall see, only the neuronal population with AHP can trigger spontaneous switching between Up and Down states while the other one, without AHP, is at the origin of the *α*-oscillations in the Up state. We also investigate the role of synaptic noise and model the effect of propofol with an excitatory current on inhibitory neurons.

## Results

### EEG reveals the dynamics of the *α*-band during general anesthesia

**Fig 1:**
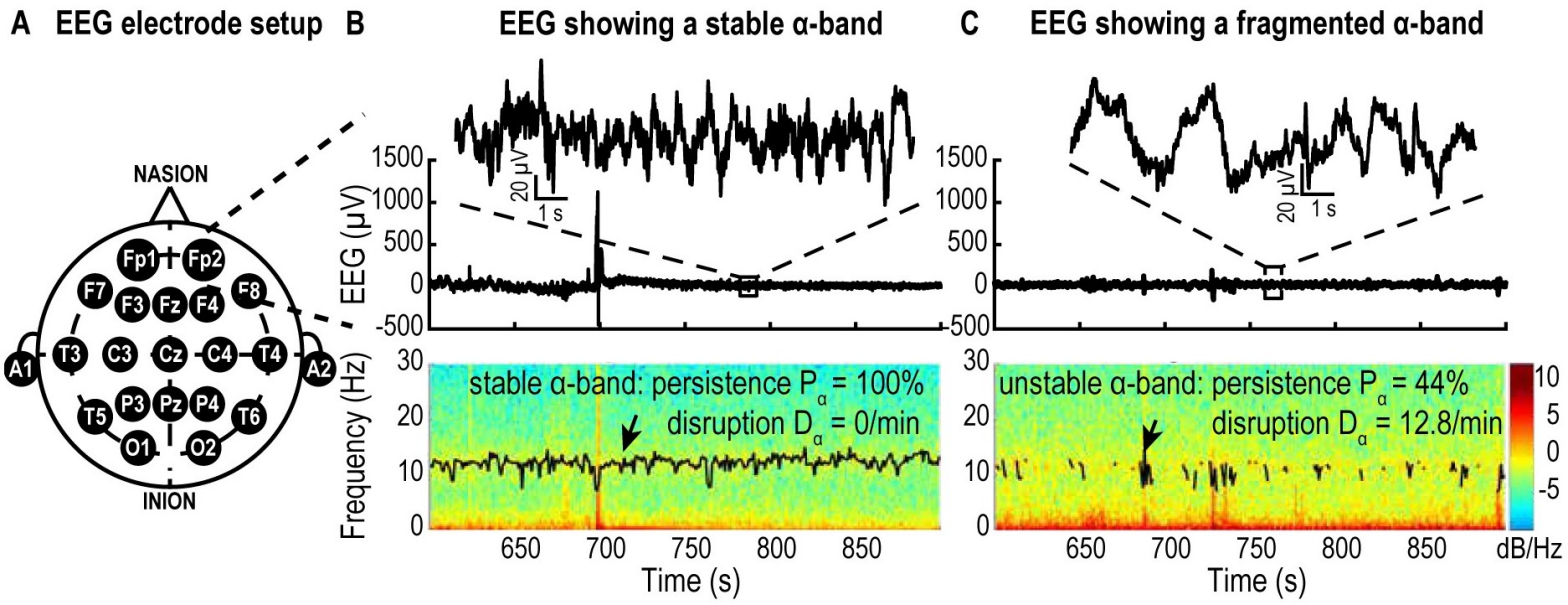
EEG recordings in patients during general anesthesia. A: Schematic of EEG electrode setup on a patient’s head. B: Upper: Time recordings showing the EEG (inset: 5 s). The EEG signal is composed of multiple bands as shown in the spectrogram (lower panel) composed of two major bands: the *δ*-band (0–4Hz) and the *α*-band (8–12Hz) tracked by its maximum (black curve) revealing the persistence of the *α*-band during anesthesia. The color indicates power in dB/Hz (see colorbar). C: Same as A for a case of fragmented *α*-band. Data from the database VitalDB [31].

General anesthesia can be monitored using EEG (Fig 1A) that often reveals a stable *α*-band which persists over time (Fig 1B). When anesthesia involves the propofol agent, it activates the inhibitory neurons, resulting in the emergence of a stable *α*-band. Increasing propofol leads to a deeper anesthesia characterized by transient removal of the *α*-band (Fig 1C) leaving a dominant *δ*-band in the spectrum. *α*-band disappearance can be quantified by two parameters, defining a fragmentation level which accounts for the percentage of presence (persistence level) *P*_*α*_ of the *α*-power over a period of time and the number of disruptions *D*_*α*_ in the power band (see Methods). In the remaining part of this manuscript we shall develop a mean-field model based on synaptic properties to study this emergence and fragmentation.

### Single neuronal population can exhibit *α*-oscillations or slow switching between Up and Down states

#### Short-term depression and facilitation generates locked *α*-oscillations

**Fig 2:**
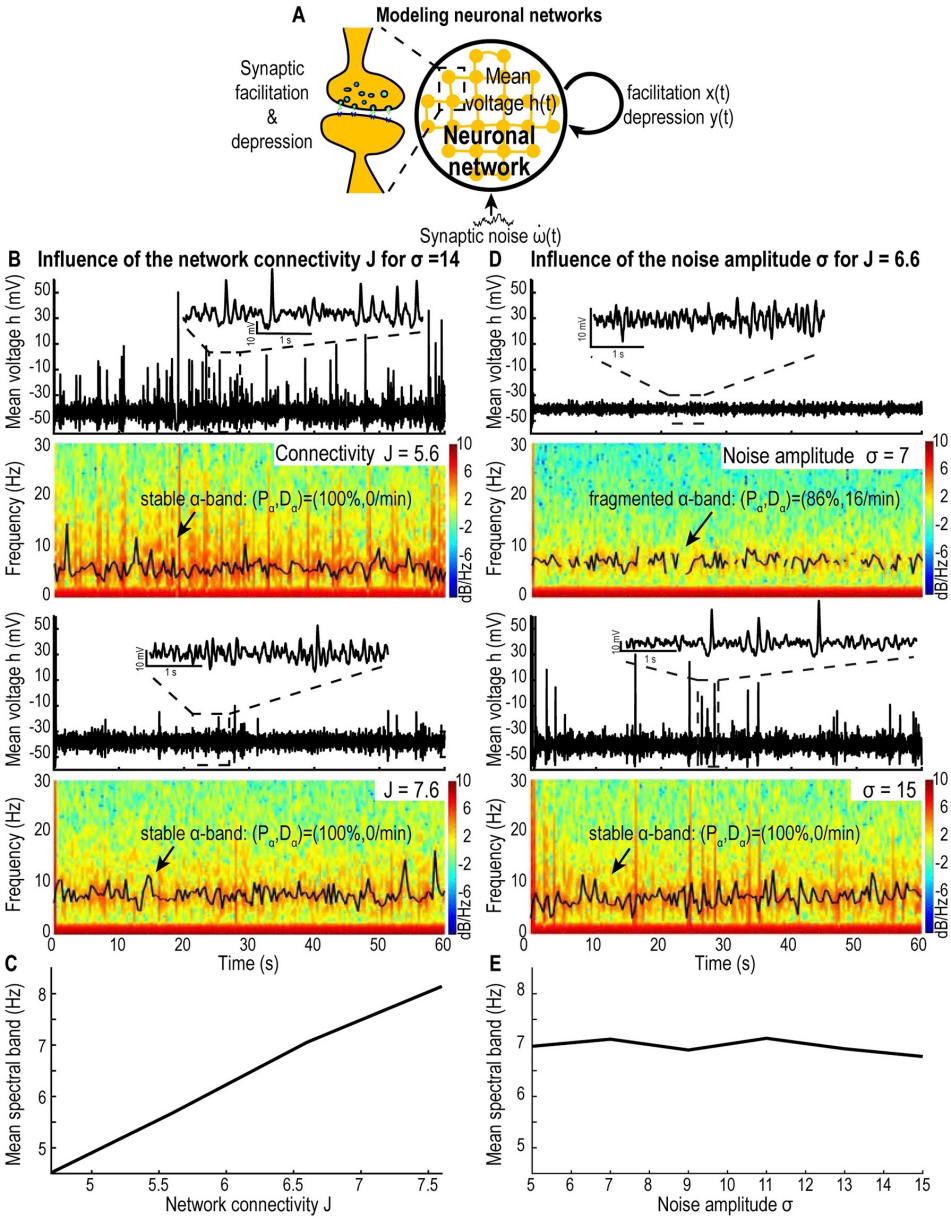
Effect of network connectivity *J* and noise amplitude *σ* on model (3) without AHP. A: Schematic of the facilitation-depression model (3). B: Time-series and spectrograms of *h* (60s simulations) with peak value of the dominant oscillatory band (black curve) for *J* = 5.6 (upper) and 7.6 (lower). C: Mean value of the dominant oscillatory frequency for *J* ∈ [4.8, 7.8]. D: Time-series and spectrograms of *h* (60s simulations) peak value of the dominant oscillatory band (black curve) for *σ* = 7 (upper) and 15 (lower). E: Mean value of the dominant oscillatory frequency for *σ* ∈ [5, 15]. Synaptic plasticity timescales: *τ* = 0.01s, *τ*_*r*_ = 0.2s and *τ*_*f*_ = 0.12*s*.

To analyze changes between a persistent *α* and a *δ*-band, we develop mean-field models of neuronal networks based on short term synaptic plasticity (Fig 2A). The first model consists of one excitatory neuronal network in which neurons are sufficiently connected so that they exhibit a synchronized activity. In this context, the average activity of the population represents each neuron’s activity. We describe this neuronal network by three variables: the mean voltage *h*, the synaptic facilitation *x* and the depression *y*, resulting in a stochastic dynamical system (see Methods, Eq 3) showing bi-stability. The dynamics presents a first attractor corresponding to the Down state (hyperpolarized, low frequency oscillations) and a second attractor associated with the Up state (depolarized, high frequency oscillations). One fundamental parameter of the model (first equation in 3) is the connectivity level *J*, which quantifies the average number of connections (synapses) between neurons in the population, that we shall vary (Fig 2B). We found that such a system can generate a dominant oscillatory band where the peak value is an increasing function of the connectivity *J* (Fig 2C). In the present scenario the network dynamics can be locked into an Up state and then the dominant oscillation frequency is generated by the imaginary part of the eigenvalues at the Up state attractor (see paragraph Origin of oscillations in the Up state in Methods for details). Sustaining these oscillations requires an additive noise, otherwise the dynamics would converge to the Up state attractor. This result shows that the persistent oscillations are the consequence of the noise and of the synaptic properties as well as the biophysical parameters (S1 Table). Changing the synaptic properties can lead to a fragmentation and disappearance of the band so that most of the remaining spectral energy is located in the *δ*-band, as quantified by the spectral edge frequency at 95% (SEF95, S1 Fig). In addition, varying the noise amplitude allows to either fragment the band (Fig 2D, *σ* = 7) or to increase its power and persistence (Fig 2D, *σ* = 15) but it does not affect the value of the peak of the dominant oscillation frequency (Fig 2E). Indeed, similarly to the EEG analysis (Fig 1), we quantified the fragmentation level for different noise amplitudes and found that it varies from (*P*_*α*_, *D*_*α*_) = (52%, 34/*min*) for a noise amplitude *σ* = 5 to (86%, 16/*min*) for *σ* = 7 and to (100%, 0/*min*) for *σ* = 15, similar to the magnitude observed in the human EEG data. This fragmentation of the *α*-band results only from the changes in the noise amplitude and can occur even though the neuronal population is locked into the Up state, suggesting that the loss the *α*-band could be independent from the switch between Up and Down states.

#### AHP with short-term depression and facilitation generates Up and Down states but no *α*-oscillations

**Fig 3:**
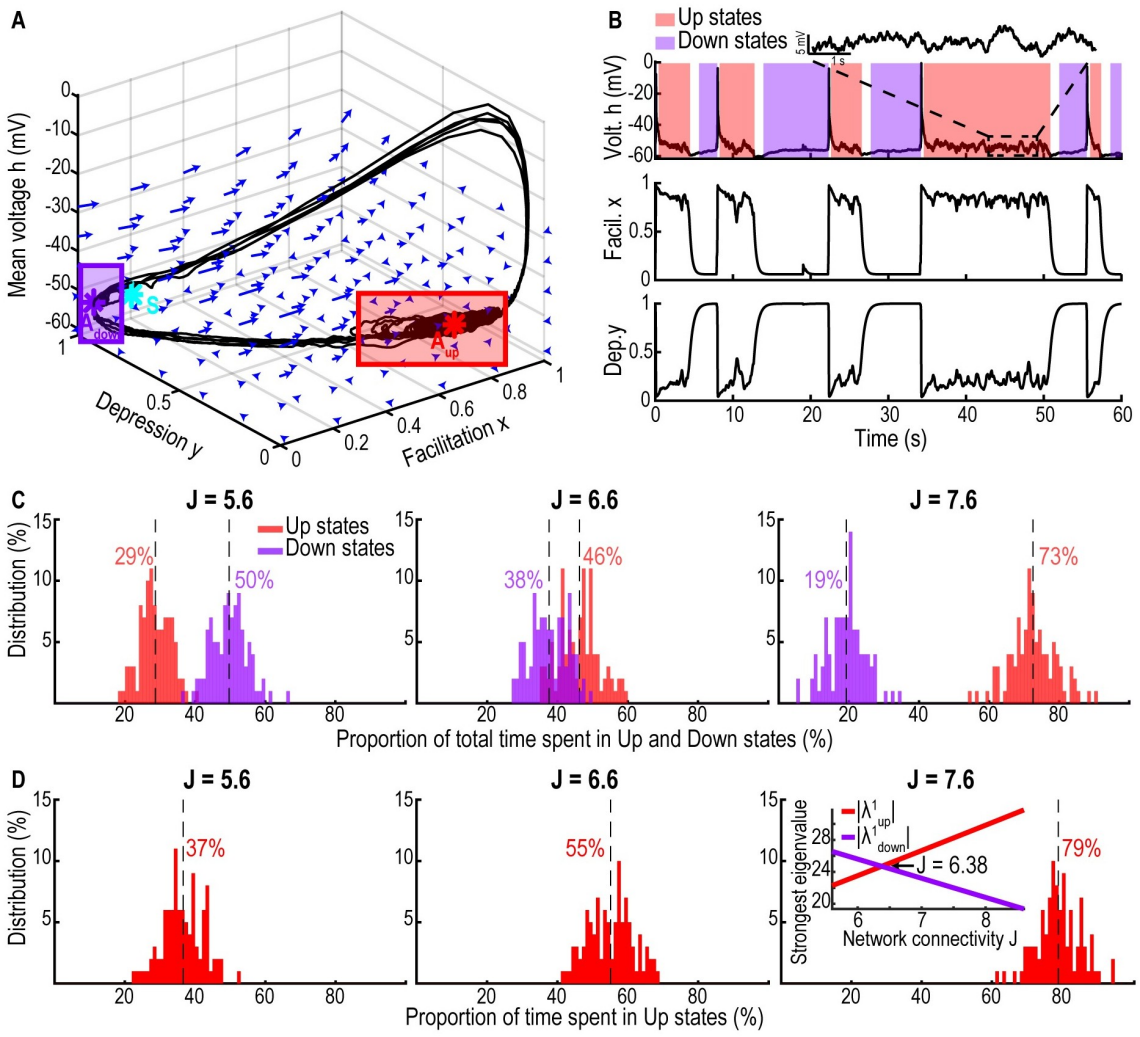
Single population exhibiting Up and Down states. A: 3D phase space of system (3) with the two attractors *A*_*Down*_ (purple) and *A*_*Up*_ (red) and the saddle-point *S* (cyan). The purple (resp. red) rectangle shows the subspace of Down (resp. Up) states. B: Time series of the mean voltage *h* (upper) showing Down (purple, resp. Up, red) states and an inset during the Up state, facilitation *x* (center) and depression *y* (lower, synaptic plasticity timescales: *τ* = 0.025s, *τ*_*r*_ = 0.5s and *τ*_*f*_ = 0.3s). C: Distributions of the fraction of time spent in Down *t*_*Down*_ (purple, resp. Up *t*_*Up*_, red) states for network connectivity *J* = 5.6 (left), 6.6 (center) and 7.6 (right) for 1000 minutes simulations. Vertical dotted lines indicate mean values. D: Distribution of the fraction of the total time spent in Up state $\frac{t_{Up}}{t_{Up}+t_{Down}}$ for the three values of *J*. Inset: strongest attractive eigenvalue of the Down state $|\lambda_{A_{Down}}^{1}|$ (purple, resp. Up state, $|\lambda_{A_{Up}}^{1}|$, red) with respect to the network connectivity *J*.

The short-term plasticity equations proposed in the previous section did not generate switching between Up and Down states, thus we decided to add the consequences of AHP to the model (see Methods). With this additional effect, it is possible to explore a larger range of neuronal dynamics. In brief, we added an AHP component to the mean-field Eq (3) presented above (see Methods). The novel dynamics exhibits bi-stability characterized by random transitions between Up and Down states Fig 3(A) and 3(B). Contrary to the system without AHP, in the Up state the system does not exhibit a dominant oscillatory band other than *δ* (S2 Fig). Interestingly, by increasing the network connectivity *J*, the fraction of time spent in the Up state can be tuned: for small *J*, (here *J* = 5.6) the dynamics spends 37% of the time in the Up state, while for *J* = 7.6 the fraction increases to 79% (Fig 3C and 3D) and S2(A) and S2(B) Fig. Finally, by increasing the noise amplitude, we observe more frequent switches between Up and Down states S2(C) and S2(D) Fig. To conclude, this model recapitulates switching between Up and Down states but cannot generate a stable *α*-band.

#### External stimulation in Up states cannot destabilize the oscillation rhythm

Adding an input current on the mean voltage *h* during the Up states simulates a situation where the observed network projects an excitatory input on a second network that would send a positive feedback when activated. The second network would only get activated by such stimuli when the first (observed) network is in the Up state. In previous studies with a 2D model (modeling only the firing rate and depression) we showed that such stimulus stabilizes the Up state [24]. Here we ran simulations for the cases with and without AHP where we added a constant input current only when the system was in the Up state (S3 Fig). In the case without AHP, the dynamics stays locked in the Up state S3(A) Fig, even in the case of a negative feedback current (upper) and the amplitude of the current *I*_*Up*_ does not affect the peak value of the oscillatory band (S3B Fig). In the case with AHP the dynamics is not changed either: the dynamics switches between Up and Down states S3(C) Fig and the proportion of time spent in either Up or Down state is not affected by the value of the current *I*_*Up*_ S3(D) Fig.

### Effect of inhibition on an excitatory neuronal network with and without AHP

**Fig 4:**
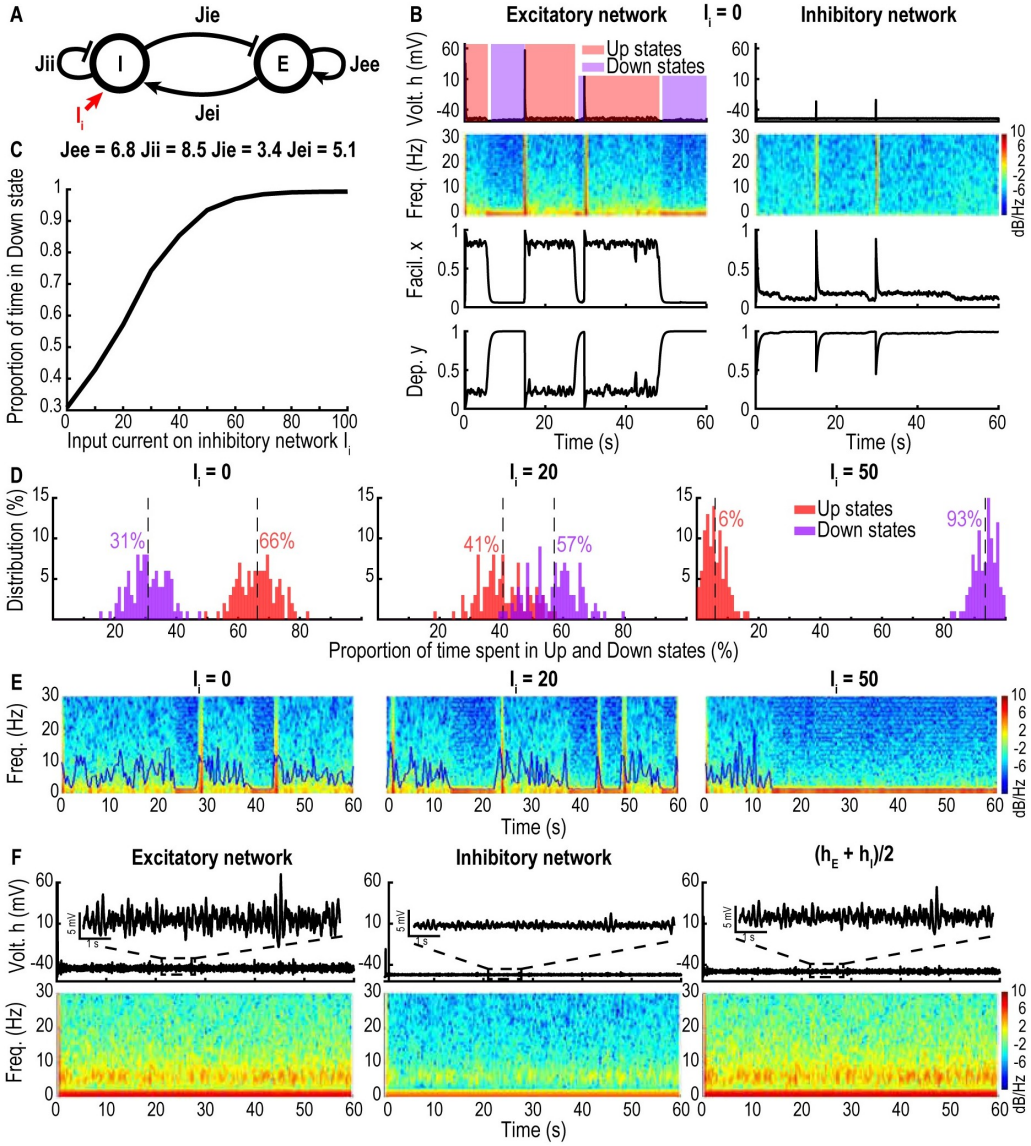
Excitatory-inhibitory network. A: Schematic of connectivity between the excitatory (E) and inhibitory (I) networks. B: Time series of the excitatory with AHP (left) and inhibitory (right) networks with the spectrograms of *h* for *J*_*EE*_ = 6.8, *J*_*II*_ = 8.5, *J*_*IE*_ = 3.4, *J*_*EI*_ = 5.1, *τ* = 0.01s, *τ*_*r*_ = 0.5s, *τ*_*f*_ = 0.3s and *I*_*i*_ = 0. C: Fraction of time spent in the Down state by the excitatory network with respect to *I*_*i*_ ∈ [0, 100]. D: Distributions of the fraction of time spent in the Down (purple, resp. Up, red) state by the excitatory network for *I*_*i*_ = 0 (left), 20 (center) and 50 (right). E: Spectrograms (60s simulations) of *h*_*E*_ for for *I*_*i*_ = 0 (left), 20 (center) and 50 (right) with SEF 95 (blue line). F: Time series of the mean voltage *h* (upper) and spectrograms (lower) for the case where the excitatory network does not have AHP and with the timescales *τ* = 0.01s, *τ*_*r*_ = 0.2s, *τ*_*f*_ = 0.12s, showing an *α*-band.

To explore the range of oscillatory behaviors, we connected an inhibitory neuronal network to an excitatory one with or without AHP (see Methods, Eq 5). We also added a constant input current *I*_*i*_ on the inhibitory population (Fig 4A).

In the case where the excitatory network has AHP the dynamics exhibit switching between Up and Down states (Fig 4B). Interestingly, by increasing the current *I*_*i*_ we can modulate the fraction of time spent in Up state by the excitatory system (Fig 4C and 4D). Furthermore, when the value of the current *I*_*i*_ injected to the inhibitory population reaches the threshold *I*_*i*_ ≥ 60, the excitatory population is locked in the Down state, therefore silencing the network (Fig 4C and 4D right panel). However, independently of the value of *I*_*i*_, the Up state does not show any persistent *α*-oscillations (Fig 4E). Finally, when connected to an excitatory population without AHP, the inhibitory network cannot induce switching between Up and Down states, and thus the dominant *α*-band generated by the excitatory population in the Up state remains stable (Fig 4F).

### Coexistence of Up and Down states and *α*-oscillations in two excitatory and one inhibitory networks

**Fig 5:**
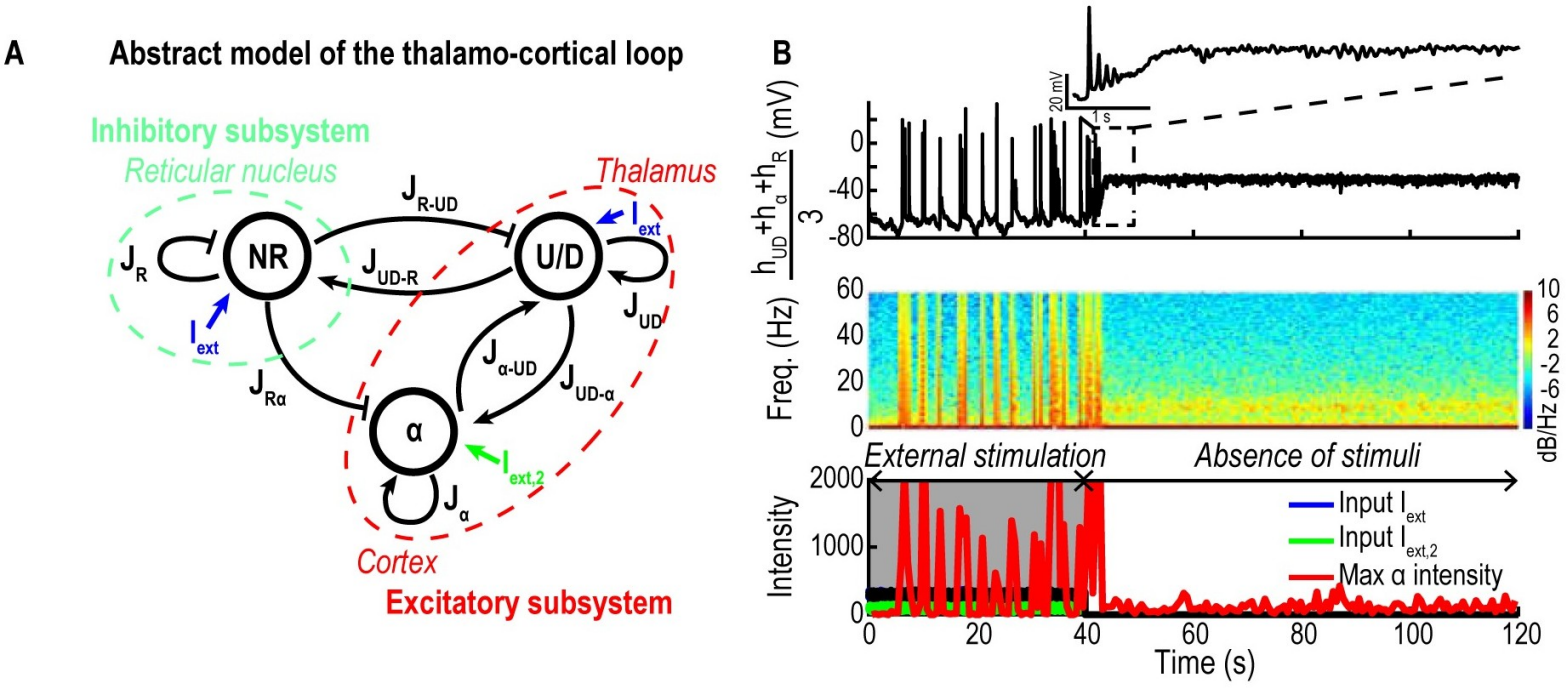
Emergence of the *α*-band following external stimuli suppression. A: Connectivity matrix between the two excitatory *α*, *U*/*D* and inhibitory (*NR*) network, with the external inputs *I*_*ext*_ (blue) and *I*_*ext*,2_ (green). B: Time-series for the mean of the three populations voltage (*h*_*α*_ + *h*_*UD*_ + *h*_*R*_)/3 (upper), spectrogram (center) and maximum value of *α* intensity (red) and inputs *I*_*ext*_ (blue and green, lower). The timescale parameters for *U*/*D* are *τ* = 0.025s, *τ*_*r*_ = 0.5s, *τ*_*f*_ = 0.3s; for *NR* and *α*: *τ* = 0.07s, *τ*_*r*_ = 0.14s, *τ*_*f*_ = 0.086s, *σ*_*UD*_ = *σ*_*R*_ = 6.25 and *σ*_*α*_ = 1.5.

To define the conditions under which the Up and Down states can coexist with an *α*-band, we coupled two excitatory networks with an inhibitory network (see Methods, Eq 6). With such topological organization, we explored whether *α*-oscillations could be generated as described for the thalamo-cortical loop [7, 13]. In our model, the thalamo-cortical excitatory subsystem is composed of two populations: 1) *α* and 2) *U*/*D* networks connected by reciprocal connections (Fig 5A). They receive an inhibitory input from the inhibitory subsystem *NR*. The *NR* component sends reciprocal connections to the *U*/*D* component and can also be activated by an external stimulation *I*_*i*_. We study the mean of the three voltage components as a proxy for a recorded EEG. During general anesthesia with propofol, increasing the dose leads to a fragmentation and transient disappearance of the *α*-band. To assess under which conditions this phenomenon could be reproduced in the model, we followed the same protocol: we first investigated the effect of switching off all external stimuli, and then by increasing an injected current to the inhibitory neuronal component, we simulated an increase of the propofol concentration.

#### Spontaneous emergence of *α*-oscillations after the suppression of external stimuli

To mimic the loss of consciousness at the start of a general anesthesia, we switched off external sensory inputs in the model. To account for this transition, we first added external stimuli modeled as excitatory input currents *I*_*ext*_ = 300 + 20*ξ* (resp. *I*_*ext*,2_ = 100 + 20*ξ*) where *ξ* is a Gaussian white noise of mean 0 and variance 1. We applied *I*_*ext*_ and *I*_*ext*,2_ to the three components of the model (Fig 5A, blue and green) for the first 40 seconds of the simulation. To model the loss of consciousness at the beginning of anesthesia, we set the external stimuli *I*_*ext*_ and *I*_*ext*,2_ to zero for the rest of the simulation (Fig 5B). We found no dominant oscillatory band in the spectrogram during wakefulness (*I*_*ext*_ > 0 from 0 to 40s). However, after external inputs suppression (*I*_*ext*_ = 0) during the interval 40 to 120s, the network stabilizes in the Up state and a dominant *α*-band emerges (Fig 5B). Here external stimuli are modeled by direct inputs including a noise term *ξ* that accounts for the variations in the sensory inputs. Note that we could have obtained a similar result with constant values of *I*_*ext*_ = 300 (resp. *I*_*ext*,2_ = 100). However, the noise term $\sigma_{x}\dot{\omega_{x}}$ in equation for *h*_*x*_ in the three populations *x* ∈ {*U*/*D*, *α*, *R*} is essential for the emergence of the *α*-band. Without this noise term, the dynamics would stay trapped at the attractor *A*_*Up*_ with no oscillations. To conclude, the present simulations suggest that the *α*-band emerges as a stable state once the external stimuli are suppressed. This behavior results from the interactions between the three networks.

#### External inputs on the inhibitory network modulate switching between Up and Down states

**Fig 6:**
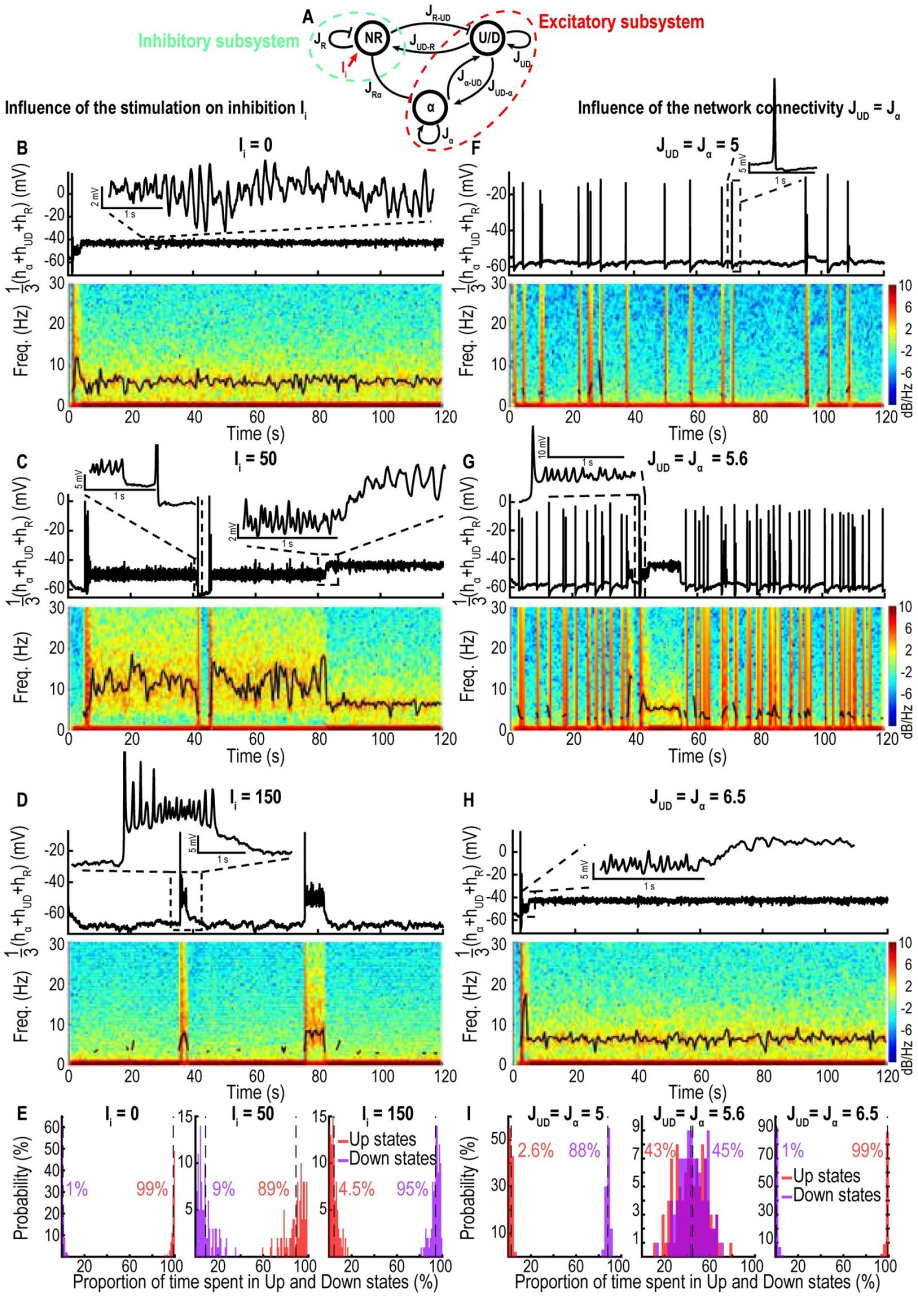
Three compartment model exhibiting Up-Down states and *α*-band in the Up state. A: Schematic of connectivity between the two excitatory (*α*, *U*/*D*) and inhibitory (*NR*) components. B-D: Time-series of the sum of the three populations voltage *h*_*α*_ + *h*_*UD*_ + *h*_*R*_ (upper) and spectrograms (lower) with position of maximum of the oscillatory band (black) for *J*_*UD*_ = *J*_*α*_ = 6.5 and *I*_*i*_ = {0, 50, 150}. E: Fraction of time spent in the Up and Down states for the three values of *I*_*i*_. F-H: Time-series of *h*_*α*_ + *h*_*UD*_ + *h*_*R*_ (Upper) and spectrograms (lower) for *I*_*i*_ = 0 and *J*_*UD*_ = {5, 5.6, 6.5}. I: Fraction of time spent in the Up and Down states for the three values of *J*_*UD*_ = *J*_*α*_. Timescales for *U*/*D*: *τ* = 0.025s, *τ*_*r*_ = 0.5s, *τ*_*f*_ = 0.3s; for *NR* and *α*: *τ* = 0.005s, *τ*_*r*_ = 0.2s, *τ*_*f*_ = 0.12s.

To better characterize switching between Up and Down states, we decided to investigate the role of inhibitory inputs. To this end, we increased the inhibitory input current *I*_*i*_ (Fig 6A) and estimated the fraction of time the networks spend in Up and Down states. For *I*_*i*_ = 0, we found that the dynamics is characterized by a large proportion of time spent in the Up state (99%), showing persistent *α*-oscillations (Fig 6B). If the transition from Up to Down is characterized by a disappearance of the *α*-band, the transition from Down to Up comes with a burst which can either lead to the emergence of an *α*-band or a return to the Down state. By increasing *I*_*i*_ from 0 to 50 and 150, we also found that the fraction of time spent in Up states decreases from 99% to 89% and then to 4.5% Fig 6(C)–6(E). During this process, each network had a different contribution to the EEG. The *U*/*D* component with AHP shows a fragmented and weak *α*-band, while the inhibitory network does not exhibit any particular oscillatory band. Finally, the *α* component without AHP exhibits a strong dominant *α*-band S4(A) and S4(B) Fig.

To study the impact of the network connectivity on the emergence of a dominant band, we varied together the intrinsic connectivities *J*_*UD*_ = *J*_*α*_ of both excitatory networks Fig 6(F)–6(H). We found that a small connectivity *J*_*UD*_ = *J*_*α*_ = 5 is associated with a large number of Down states (88%) and that transient bursts rarely lead to a stable *α*-band (Fig 6F). By increasing the connectivity *J*_*UD*_ = *J*_*α*_ to 5.6 and 6.5, the fraction of Up states increases to 43% and 99% respectively (Fig 6I) leading to stable Up states associated with a persistent *α*-band. To conclude, the amplitude of inhibitory inputs as well as the connectivity level are fundamental parameters that modify the proportion of Up states and thus the persistence of the *α*-oscillations.

#### Transient responses of the thalamo-cortical model to step and stairs inputs

**Fig 7:**
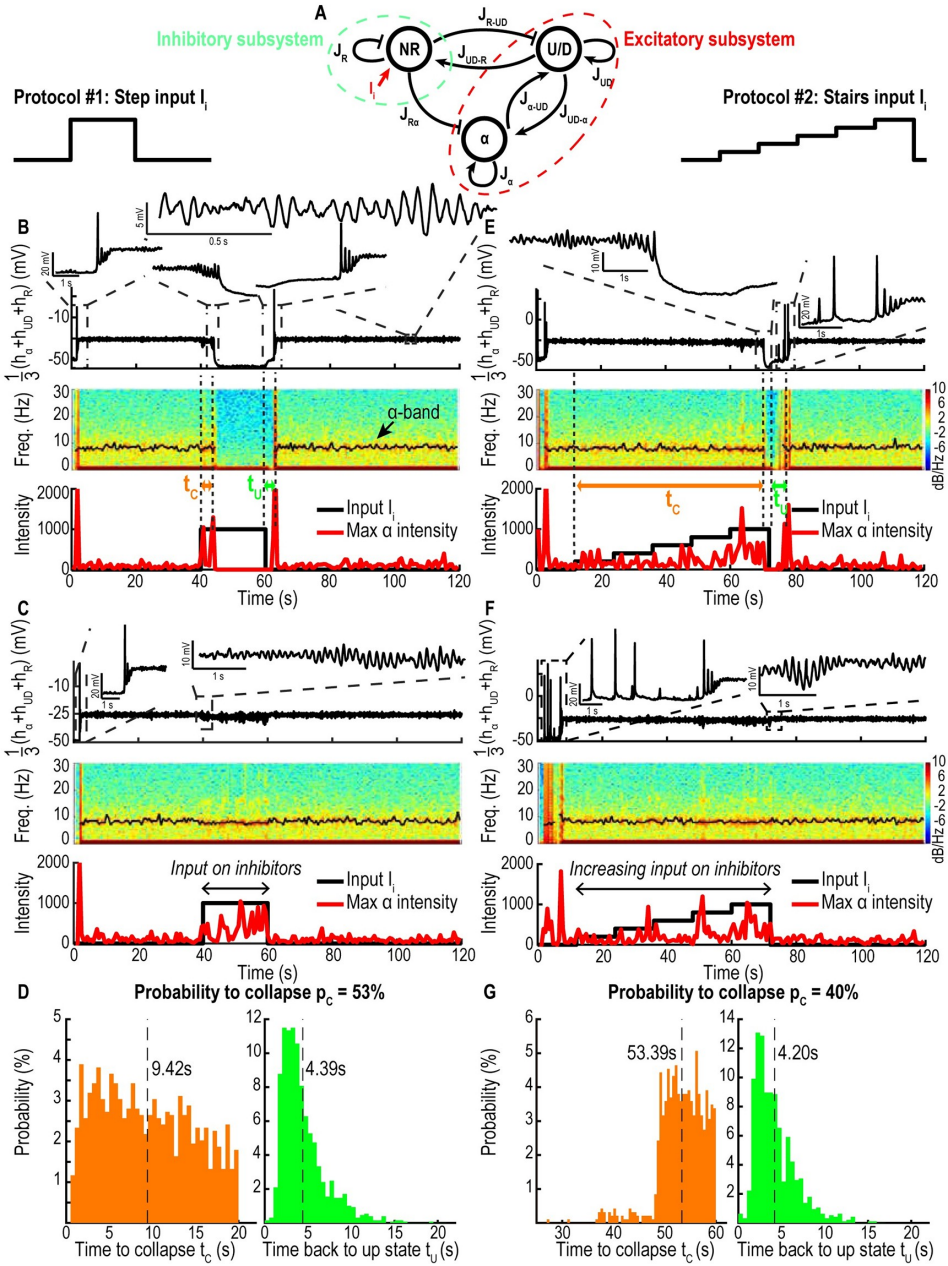
Cases of collapse of the *α*-band. A: Schematic of connectivity between the two excitatory (*α* and *U*/*D*) and inhibitory (*NR*) networks. B-C: Time-series of the sum of the three populations voltage *h*_*α*_ + *h*_*UD*_ + *h*_*R*_ (upper), spectrogram (center) with position of maximum of the *α*-band (black, collapsing (B) and persistent (C)), maximum value of *α* intensity (red) and input *I*_*i*_ (black, lower) for a step input. D: Distributions of the delay *t*_*C*_ between the beginning of stimulation and the collapse (orange) and delay between the end of the stimulation and the moment the reapparition of the *α*-band (green). E-G: Same as B-D for a stairs input. Timescales for *U*/*D*, *α* and *NR*: *τ* = 0.005s, *τ*_*r*_ = 0.1s, *τ*_*f*_ = 0.06s.

To study, the possible thalamo-cortical response to a bolus of the hypnotic agent propofol, we simulated two protocols: 1) a step input (protocol 1) and 2) a stairs input (protocol 2) as shown in Fig 7A.

To analyze the response to protocol 1, we ran simulations with *N* = 2500 iterations lasting *T* = 2min, where a strong injection is delivered by simulating a positive input current *I*_*i*_ = 1000 on the inhibitory network (*NR*) lasting *t*_*i*_ = 20s (Fig 7B and 7C). To quantify the response we focused on two parameters: 1) the duration *t*_*C*_ after which the *α*-band disappears after the step function begins. 2) The duration *t*_*U*_ after which the *α*-band reappears after the end of the step function. Interestingly, for some realizations the *α*-band does not disappear (Fig 7C), we thus characterized this effect by the collapse probability *p*_*C*_. We found a probability value of *p*_*C*_ = 53% and a duration *t*_*C*_ = 9.42 ± 5.36s while *t*_*U*_ = 4.39 ± 2.58s (Fig 7D). The histogram for *t*_*C*_ is characterized by an abrupt decay at 20s confirming that *α*-band suppression can only occur during the stimulation period. However, the time *t*_*U*_ is dominated by an exponential decay, a classical feature of dynamical systems driven by noise.

Moreover, each network has a different contribution to the EEG: the *U*/*D* excitatory network with AHP shows an *α*-band while the inhibitory network *NR* only exhibits weak power in the slow *δ*-wave region for frequencies ≤ 1*Hz*. Finally, the *α* excitatory component without AHP exhibits a strong dominant *α*-band S5(A) and S5(B) Fig.

To analyze the effect of a slower increase of the input (stairs function, protocol 2) we ran simulations with *N* = 2500 iterations lasting *T* = 2min, where we simulated a stairs increase from an initial current *I*_*i*_ = 0 up to 1000 on the inhibitory network *NR*, lasting *t*_*i*_ = 60s Fig 7(E) and 7(F). The statistics of the durations *t*_*C*_ which characterizes *α*-band disappearance, *t*_*U*_ measuring its re-emergence, as well as the probability to collapse *p*_*C*_ are given by *p*_*C*_ = 40%, *t*_*C*_ = 53.39 ± 4.44s and *t*_*U*_ = 4.20 ± 2.43s (Fig 7G). Similarly, the histogram of *t*_*C*_ is characterized by an abrupt decay at 60s confirming that the suppression of the *α*-band can only occur during the stimulation period, while the histogram of *t*_*U*_ is dominated by an exponential decay. These results quantify the impact of the intensity and duration of a propofol bolus on the *α*-band.

## Discussion

We presented here a parsimonious computational approach based on coarse-grained neuronal network models in order to generate *α*-oscillations. Interestingly, a single neuronal population driven by synaptic short-term plasticity can elicit oscillations at a defined frequency, which directly depends on the value of the network connectivity: a higher connectivity generates faster oscillations (Fig 2B and 2C). Interestingly, we show here that stable *α*-oscillations result from the combination of network connectivity, the overall synaptic strength and the intrinsic biophysical properties of the network, leading to the emergence of a focus attractor, around which the mean population voltage oscillates in the phase-space S7(A) and S7(B) Fig. Moreover, we showed that spontaneous switching between Up and Down states in a single neuronal population is modulated by AHP and also that the network connectivity controls the proportion of Up vs Down states: a higher connectivity *J* results in a dominant percentage of time spent in Up states (Fig 3C and 3D). The stability of the oscillations during Up states for a population without AHP could result from the intrinsic network regulation: indeed, interactions between inhibitory interneurons and hippocampal pyramidal excitatory neurons can redistribute the firing load to maintain the oscillation frequency even when up to 25% of the synapses are deactivated [32].

**Fig 8:**
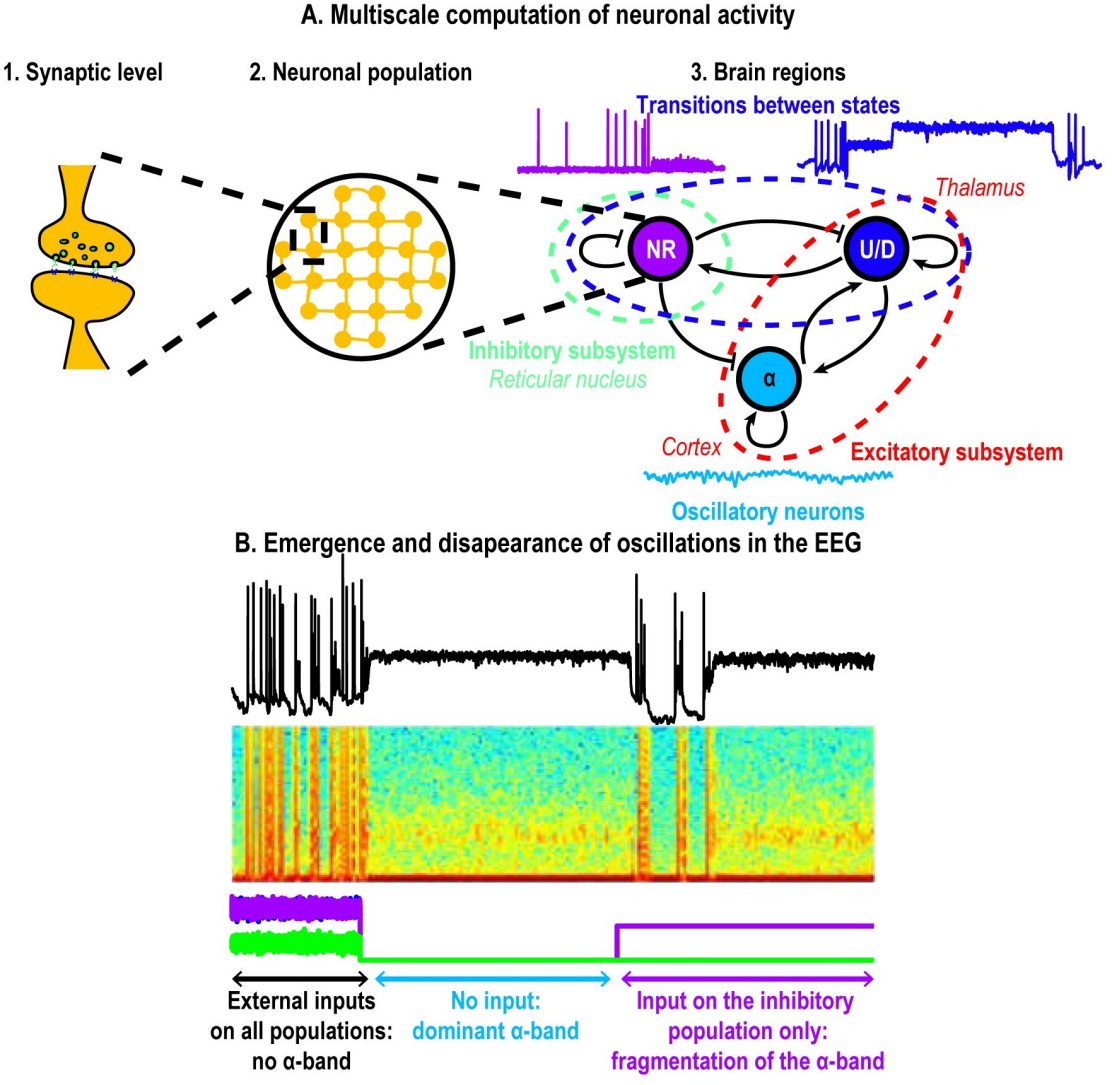
Computational principle underlying the *α*-band dynamics. A: Multiscale models 1. synaptic level: short-term facilitation and depression mechanisms, 2. neuronal population: mean voltage fluctuations and 3. interactions between different brain regions (thalamus and cortex, models 5 and 6) resulting in the different activity patterns observed in EEG at a global scale. B: *α*-band emerges in the Up-state due to noise but can be altered by external inputs.

When we added an excitatory input current on an inhibitory population coupled to an excitatory population, the proportion of time spent in Up states decreased and, after reaching a threshold value *I*_*i*_ = 60, the network became completely silenced, characterized by Down states only (Fig 4C and 4D). When coupling two excitatory populations and one inhibitory population (Fig 5A), *α*-oscillations, generated by the excitatory component without AHP, co-existed with spontaneous switching between Up and Down states induced by the excitatory population with AHP, as summarized in Fig 8. Stimulating the inhibitory population modulates directly the proportion of Up vs Down states and thus induces a fragmentation of the *α*-band (Fig 6B–6E).

Finally, we suggest that synaptic noise has two main roles on the network properties: 1) increasing the noise intensity stabilizes the *α*-band (Fig 2D) and 2) when an external stimulation is applied to the inhibitory system in a step or stairs input, the network can react with two opposite behaviors: either the network activity collapses, leading to a suppression of all oscillatory bands in the EEG or a stable persistent *α*-band emerges during the entire stimulation (Fig 7). Finally, we propose that three connected neuronal populations are sufficient to generate an *α*-oscillation that could be fragmented by increasing the inhibitory pathway, as suggested during general anesthesia [7]. The present model could be generalized to other brain rhythms to study the emergence and disappearance of other oscillations such as the *θ*-oscillations occurring during REM sleep [2, 33, 34].

### Modeling the dynamics of the *α*-band

For several decades, the origin of the *α*-band [14] had remained unclear. Early modeling efforts using the Hodgkin-Huxley framework [30] suggested a key role of ionic currents such as sodium, potassium currents, low threshold calcium, AHP and synaptic currents (GABAs, AMPA) that could reproduce various patterns of oscillations [19] as well as initiation, propagation and termination of oscillations (see also [35]). By varying the GABA conductances other models have reproduced the dominant *α*-oscillation observed in propofol anesthesia [20, 21]. Indeed GABA conductances regulate the firing frequencies and synchronization of pyramidal neurons [36]. In contrast to conductance based models, we used here short-term synaptic plasticity driven by noise and found that the *α*-band can be generated only when the mean voltage reaches the Up state, suggesting that the ionic mechanisms are not necessary to generate the *α*-band, but contributes to the termination of the Up states. Furthermore, adding an input current to the inhibitory population allows to modulate the transitions between Up and Down states (Figs 4C, 4D and 6B–6E). In addition, we found that the *α*-band can be stabilized by increasing the noise amplitude, while the peak frequency of the *α*-band was unchanged (Fig 2D–2E). Thus, we propose that the synaptic noise could be responsible for the stabilization of the *α*-band, as quantified by the present fragmentation analysis (Fig 2D–2E). Finally, we reported a second mechanism responsible for an *α*-band fragmentation which is associated with the transition between Up and Down states (Fig 6).

Interestingly, in the medical context, the *α*-band is persistent in young subjects and becomes sparser with age [37]. Possibly, a higher neuronal activity (in younger subjects) leads to higher extracellular potassium which, in turn, increases the synaptic noise [38]. Another possible mechanism for fragmenting the *α*-band could involve the metabolic pathway, when the ATP coupled to the sodium concentration is decreased: during a burst, a high sodium concentration depletes ATP that deregulates the potassium current and thus leads to a phase of iso-electric suppression [11].

### Up and Down states and the *α*-band

Neuronal networks exhibit collective transitions from Up to Down states [15, 16, 22]. This transitions from Up to Down states are responsible for the slow wave oscillations in the *δ* range, observed in deep sleep as well as anesthesia and probably originates from the thalamic network [16–18]. Previous modeling efforts have accounted for this phenomenon using single compartment neuron models with fast and slow currents responsible for neuronal spiking and slow modulation of the membrane voltage, respectively [39]. It was also shown that increasing hyperpolarization in a Hodgkin-Huxley type model, including calcium and hyperpolarizing currents [30] that was previously used to reproduce waxing and waning oscillations around 4Hz, could reduce the oscillation frequency [40]. Our approach here is thus in agreement with the previous theory even though we use a different modeling strategy. Indeed, we reported that the *α*-oscillation is only generated when the neuronal ensemble is in the Up state. We could not generate simultaneously in a single neuronal population both the *α*-oscillation and the Up-Down states transitions. Rather we needed a minimum of two coupled excitatory neuronal populations, one of them representing the thalamic neurons (Fig 5A U/D network) the function of which was to trigger the transitions between Up and Down states (Fig 6). Furthermore, we have shown that this neuronal population needs to account for hyperpolarization in order to trigger these Up-Down transitions (Fig 3) as also seen with classical Hodgkin-Huxley type models [30]. We reported here that the fraction of time spent in Up and Down states depends on the level of synaptic connectivity (Figs 3C, 3D and 6F–6I). However, by adding an inhibitory network, we were able to modulate the proportion of time spent in Up vs Down states by changing the input stimulation current (Figs 4C, 4D and 6B–6E) on this inhibitory population without varying the connectivity. This mechanism is feasible because the inhibitory input on both excitatory populations allows to destabilize the Up state and thus increase the transitions to Down state modulating the overall fraction of time spent in the Up state. To conclude, in the extreme case where the Down states are dominant, the overall voltage dynamics could resemble iso-electric suppressions, but this dynamics does necessarily rely on a metabolic stress [41, 42].

### How to interpret the *α*-band during general anesthesia?

During general anesthesia, the physiological mechanism leading to the emergence of the *α*-band shortly after propofol injection remains unclear [7, 43]. Possibly, during wakefulness, the amount of external stimuli suppresses the emergence of *α*-oscillations [2]. When the external stimuli ceases with propofol injection, the *α*-oscillation could become dominant (Fig 5B). The present model suggests that the initial state represents an already anesthetized brain where the neuronal networks do not receive any external stimuli, leading to the spontaneous emergence of the *α*-band.

General anesthesia needs to be sustained over the whole course of a surgery and thus controlling the optimal anesthetic injection to prevent cortical awareness or a too deep anesthesia remains a difficult problem [8, 37]. Population models such as the one presented here could be used to test different activation pathways of anesthetic drugs. The present model accounts for the appearance an iso-electric suppression [44] in the EEG of the order of a few seconds (Fig 7B and 7E) induced by increasing transiently the hypnotic. We predicted here that the fragmentation of the *α*-band results from a shift between Up and Down states dominance that could be tested with in vivo experiments. It would be interesting to further account for longer term consequences of an anesthetic input (several minutes). Indeed, the causality between *α*-suppressions and burst-suppressions [8] remains unexplained, suggesting that this relation could involve other mechanisms than the ones we modeled here based on synaptic plasticity, AHP and network connectivity.

## Methods

### Temporal fragmentation of an oscillatory band

We define here a measure for the persistence in time of the *α*-band. First we detect the peak spectral value *S*_*α*_(*t*) of the spectrogram as the highest power value in the extended range *α*_*min*_ = 4 − *α*_*max*_ = 16 Hz. When the condition *S*_*α*_(*t*) > *T*_*α*_ is satisfied, we consider that the band is present and attribute a value *x*_*pr*_(*t*) = 1, otherwise *x*_*pr*_(*t*) = 0. When the time interval between 0 and *T* is divided into N bins at times *t*_*k*_, the persistence of the *α* band is defined by

$$\begin{array}{c} P_{\alpha }=\frac{1}{N}\sum_{k=1}^{N}x_{pr}\left(t_{k}\right). \end{array}$$ (1)

The persistence level *P*_*α*_ measures the proportion of time where the *α*-band is present.

To further quantify the fragmentation level, we introduce the disruption number *D*_*α*_ that counts the number of times per minute where the peak spectral value *S*_*α*_(*t*) goes under the threshold *T*_*α*_.

$$\begin{array}{c} D_{\alpha }=\frac{1}{T}\sum_{k=1}^{N-1}x_{pr}\left(t_{k}\right)\left(1-x_{pr}\left(t_{k+1}\right)\right). \end{array}$$ (2)

We call the fragmentation level the pair *F*_*α*_ = (*P*_*α*_, *D*_*α*_) (S6 Fig). For the human EEG data (Fig 1), we used a bin size *w* = 0.5*s* and a threshold value *T*_*α*_ = 1.5*dB*. For the simulated data, we use the same bin size and *T*_*α*_ = 10*dB*.

### Modeling a single neuronal population based on short-term depression-facilitation

For a sufficiently well connected ensemble of neurons, we use a mean-field system of equations to study bursting dynamics, AHP and the emergence of Up and Down states. This stochastic dynamical system consists of three equations [23, 26, 29] for the mean-field variable *h*, the depression *y*, and the synaptic facilitation *x*:

$$\begin{array}{c} \begin{array}{ccc} \tau_{0}\dot{h} & = & -\left(h-T_{0}\right)+Jxy{\left(h-T_{0}\right)}^{+}+\sqrt{\tau_{0}}\sigma \dot{\omega }\\ \dot{x} & = & \frac{X-x}{\tau_{f}}+K\left(1-x\right){\left(h-T_{0}\right)}^{+}\\ \dot{y} & = & \frac{1-y}{\tau_{r}}-Lxy{\left(h-T_{0}\right)}^{+}, \end{array} \end{array}$$ (3)

where *h*^+^ = *max*(*h*, 0) is the population mean firing rate [24]. The term *Jxy* reflects the combined synaptic short-term dynamics with the network activity. The second equation describes facilitation, and the third one depression. The parameter *J* accounts for the mean number of synaptic connections per neuron [23, 45]. We previously distinguished [26] the parameters *K* and *L* which describe how the firing rate is transformed into synaptic events that are changing the duration and probability of vesicular release respectively. The time scales *τ*_*f*_ and *τ*_*r*_ define the recovery of a synapse from the network activity. We account for AHP with two features: 1) a new equilibrium state representing hyperpolarization after the peak response of the burst 2) two timescales for the medium and slow recovery to the resting membrane potential to describe the slow transient to the steady state. Finally, $\dot{\omega }$ is an additive Gaussian noise and *σ* its amplitude, representing fluctuations in the mean voltage.

In the case of a neuronal network that does not exhibit AHP the resting membrane potential is constant *T*_0_ = 0 and *τ*_0_ = *cst* ∈ [0.005, 0.025]s. However, for a population showing AHP after the bursts, the resting membrane potential *T*_0_ and the recovery time constant *τ*_0_ of the voltage *h* are defined piece-wise as follows S8(A) and S8(B) Fig:

- *τ*_0_ = *τ* and *T*_0_ = 0 in the subspace Ω_*fast*_ = {*y* > *Y*_*AHP*_ and *h* ≥ *H*_*AHP*_}, which represents the fluctuations around the resting membrane potential during the down state and the burst dynamics.
- *τ*_0_ = *τ*_*mAHP*_ and *T*_0_ = *T*_*AHP*_ < 0 in the subspace $\Omega_{mAHP}=\{y<\frac{1}{1+Lx(h-T_{0})}\left(\Leftrightarrow \dot{y}>0\right)$ and *y* < *Y*_*h*_}. This part of the phase-space defines the moment when the hyperpolarizing currents at the end of the burst become dominant and force the voltage to hyperpolarize.
- *τ*_0_ = *τ*_*sAHP*_ and *T*_0_ = 0 in the subspace $\Omega_{sAHP}=\{y<\frac{1}{1+Lx(h-T_{0})}$ and (*Y*_*AHP*_ < *y* or *h* < *H*_*AHP*_)}, which represents the slow recovery to resting membrane potential.

The threshold parameters defining the three phases are *Y*_*h*_ = 0.5, *Y*_*AHP*_ = 0.85 and *H*_*AHP*_ = −7.5. In this study, we varied the network connectivity parameter *J* ∈ [5.6, 8.6] and all other parameters are described in S1 Table.

To convert the mean-field variable *h* into a mean voltage $\tilde{h}$ in mV, we use the following conversion

$$\begin{array}{c} \tilde{h}=\frac{h-h_{min}}{h_{max}-h_{min}}A_{max}+V_{rest}, \end{array}$$ (4)

where *V*_*rest*_ = −70 mV. We identified *h*_*min*_ = −100 and *h*_*max*_ = 1200 based on numerical simulations and chose *A*_*max*_ = 200*mV* according to the classical amplitude of intracellular recordings.

### Two-populations model of the thalamo-cortical loop

To model the interactions between one excitatory *E* and inhibitory *I* neuronal network, we coupled two systems of Eq (3)) as follows:

$$\begin{array}{c} \begin{array}{ccc} \tau_{0}\dot{h_{E}} & = & -\left(h_{E}-T_{0}\right)+J_{EE}x_{E}y_{E}{\left(h_{E}-T_{0}\right)}^{+}-J_{IE}\frac{\tau_{0}}{\tau }x_{I}y_{I}{\left(h_{I}-T\right)}^{+}+\sqrt{\tau_{0}}\sigma_{E}{\dot{\omega }}_{E}\\ \dot{x_{E}} & = & \frac{X-x_{E}}{\tau_{f}}+K\left(1-x_{E}\right){\left(h_{E}-T_{0}\right)}^{+}\\ \dot{y_{E}} & = & \frac{1-y_{E}}{\tau_{r}}-Lx_{E}y_{E}{\left(h_{E}-T_{0}\right)}^{+},\\ \tau \dot{h_{I}} & = & -\left(h_{I}-T\right)-J_{II}x_{I}y_{I}{\left(h_{I}-T\right)}^{+}+J_{EI}\frac{\tau }{\tau_{0}}x_{E}y_{E}{\left(h_{E}-T_{0}\right)}^{+}+\sqrt{\tau }\sigma_{I}{\dot{\omega }}_{I}+I_{i}\\ \dot{x_{I}} & = & \frac{X-x_{I}}{\tau_{f}}+K\left(1-x_{I}\right){\left(h_{I}-T\right)}^{+}\\ \dot{y_{I}} & = & \frac{1-y_{I}}{\tau_{r}}-Lx_{I}y_{I}{\left(h_{I}-T\right)}^{+}, \end{array} \end{array}$$ (5)

where *τ*_0_ and *T*_0_ for the excitatory population can either be constant, in the absence of AHP or defined piece-wise when it is present, as already discussed for model 3. The inhibitory population is always modeled without AHP and thus *τ* is constant and *T* = 0. All other parameters are described in the central columns called “2 populations” of S1 Table.

### Thalamo-cortical loop model with three neuronal network populations

To model the thalamo-cortical loop, we connected three neuronal networks. One excitatory network driven by AHP generates the Up-Down states dynamics (referred to as *U*/*D* in Figs 5, 6 and 7). The second excitatory network is not driven by AHP and is referred as *α* in Figs 5, 6 and 7. Both networks are coupled with an inhibitory one (called *NR*), which does not exhibit any AHP. The equations extend the case of two neuronal networks presented in the previous subsection and the connectivity matrix with 9 elements is presented in S1 Table. The overall system of equations is

$$\begin{array}{c} \begin{array}{ccc} \tau_{0}\dot{h_{UD}} & = & -\left(h_{UD}-T_{0}\right)+J_{UD}x_{UD}y_{UD}{\left(h_{UD}-T_{0}\right)}^{+}+J_{\alpha -UD}\frac{\tau_{0}}{\tau }x_{\alpha }y_{\alpha }{\left(h_{\alpha }-T\right)}^{+}\\ & & -J_{R-UD}\frac{\tau_{0}}{\tau }x_{R}y_{R}{\left(h_{R}-T\right)}^{+}+\sqrt{\tau_{0}}\sigma_{UD}{\dot{\omega }}_{UD}\\ \dot{x_{UD}} & = & \frac{X-x_{UD}}{\tau_{f}}+K\left(1-x_{UD}\right){\left(h_{UD}-T_{0}\right)}^{+}\\ \dot{y_{UD}} & = & \frac{1-y_{UD}}{\tau_{r}}-Lx_{UD}y_{UD}{\left(h_{UD}-T_{0}\right)}^{+},\\ \tau \dot{h_{\alpha }} & = & -\left(h_{\alpha }-T\right)+J_{\alpha }x_{\alpha }y_{\alpha }{\left(h_{\alpha }-T\right)}^{+}+J_{UD-\alpha }\frac{\tau }{\tau_{0}}x_{UD}y_{UD}{\left(h_{UD}-T_{0}\right)}^{+}\\ & & -J_{R-\alpha }x_{R}y_{R}{\left(h_{R}-T\right)}^{+}+\sqrt{\tau }\sigma_{\alpha }{\dot{\omega }}_{\alpha }\\ \dot{x_{\alpha }} & = & \frac{X-x_{\alpha }}{\tau_{f}}+K\left(1-x_{\alpha }\right){\left(h_{\alpha }-T\right)}^{+}\\ \dot{y_{\alpha }} & = & \frac{1-y_{\alpha }}{\tau_{r}}-Lx_{\alpha }y_{\alpha }{\left(h_{\alpha }-T\right)}^{+},\\ \tau \dot{h_{R}} & = & -\left(h_{R}-T\right)-J_{R}x_{R}y_{R}{\left(h_{R}-T\right)}^{+}+J_{UD-R}\frac{\tau }{\tau_{0}}x_{UD}y_{UD}{\left(h_{UD}-T_{0}\right)}^{+}\\ & & +J_{\alpha -R}x_{\alpha }y_{\alpha }{\left(h_{\alpha }-T\right)}^{+}+\sqrt{\tau }\sigma_{R}{\dot{\omega }}_{R}+I_{i}\\ \dot{x_{R}} & = & \frac{X-x_{R}}{\tau_{f}}+K\left(1-x_{R}\right){\left(h_{R}-T\right)}^{+}\\ \dot{y_{R}} & = & \frac{1-y_{R}}{\tau_{r}}-Lx_{R}y_{R}{\left(h_{R}-T\right)}^{+}, \end{array} \end{array}$$ (6)

where *τ*_0_ and *T*_0_ for the first excitatory population *U*/*D* are defined piece-wise in part and all other parameters are given in S1 Table (right columns: “3 populations”).

### Origin of oscillations in the Up state

#### Oscillations around the Up state attractor for a neuronal population without AHP

In the absence of AHP, the focus attractor *A*_*Up*_ has two complex conjugated eigenvalues. Thus the deterministic dynamics oscillates around the point *A*_*Up*_ at a frequency

$$\begin{array}{c} 2\pi \omega_{Up}=Im\left(\lambda_{2}^{A_{Up}}\right)\Leftrightarrow \omega_{Up}\in \left[5.85,8.26\right]Hz. \end{array}$$ (7)

which corresponds to the dominant spectral band observed in Fig 2. The oscillation eigenfrequency *ω*_*Up*_ further depends on the network connectivity *J* (Fig 2B and 2C), but not on the noise amplitude (Fig 2D and 2E). Note that the noise allows to generate persistent oscillations compared to the case of the pure deterministic system.

Note that if we take *τ* = 0.025*s*, *τ*_*r*_ = 0.5*s* and *τ*_*f*_ = 0.3*s*, then the eigenvalues of *A*_*Up*_ become $\lambda_{A_{Up}}^{1}\in \left[-22.28,-31.76\right]$ and the complex-conjugate eigenvalues

$$\lambda_{A_{Up}}^{2,3}\in \left[-2.46,-5.89\right]\pm i\left[14.71,20.75\right]$$

leading to an eigenfrequency *ω*_*Up*_ ∈ [2.34, 3.30]Hz (S1 Fig) which explains the disappearance of the dominant *α*-band in this case see also S7(C) Fig.

Finally, since $|\lambda_{A_{Up}}^{1}|\gg |Re\left(\lambda_{A_{Up}}^{2}\right)|$ the dynamics is very anisotropic and the oscillations are confined in a 2D manifold S7 Fig (A.1-A.3, light red trajectories).

#### Up state stability due to multiple re-entries in its basin of attraction

To explain the locking in the Up state, we recall that the stochastic trajectories starting inside the basin of attraction of the Up state can cross the separatrix Γ and fall into the Down state. However, because the deterministic vector field of system (3) is very shallow near Γ, the additive noise on the *h* variable can push the trajectories back into the Up state, where the field is stronger, and thus the trajectory is brought back into a neighborhood of *A*_*Up*_ and continues to oscillate, as shown in S7(B) Fig (see inset).

To explain the other frequencies (than the eigenfrequency *ω*_*Up*_) observed in the spectrum of *h* S7(C) Fig, we note that when a trajectory falls back in the Up state, it can produce a longer or shorter loop depending on its initial distance to the attractor *A*_*Up*_. These oscillations between the two basins of attraction define stochastic oscillations that contribute to the spectrogram of *h*.

#### Oscillations between Up and Down state in the phase-space

For a neuronal network with an AHP component, the Up state attractor has only real negative eigenvalues S8(C) Fig, thus no oscillations are expected near the attractor. However, the presence of a slow AHP component S8 Fig (A-B pink) can push the dynamics into the Down state, as opposed to the case without AHP. Finally, in the Down state, the trajectories fluctuate with the noise until they escape. Once trajectories cross the separatrix Γ, they follow an almost deterministic path close to that of the unstable manifold of *S* S8 Fig (A-B grey) showing a long excursion in the phase-space before falling back near the attractor *A*_*Up*_. This dynamics explains the recurrent switches between Up and Down states.

## Supporting information

S1 Table Models (3) (1 population), (5) (2 populations) and (6) (3 populations) parameters. For models (5) and (6), the inhibitory population is always without AHP and excitatory populations can be with or without AHP. For model (6) *E*_1_ corresponds to the network with AHP (*U*/*D*), and *E*_2_ to the network without AHP (*α*). The parameters values are chosen in agreement with [23, 24, 26, 29, 46]. The timescales *τ*_*f*_ and *τ*_*r*_ and the facilitation and depression rates *K* and *L* have been scaled in order to obtain oscillations in the range 5 − 15Hz around the Up state attractor *A*_*Up*_. The network connectivity values *J*_*ii*_ (*i* = {*I*, *E*_1_, *E*_2_}) are adjusted (*J* = 4.21 in [29]) in order to reach a bifurcation that transforms the fixed point *A*_*Up*_ from a saddle-point in [29] to an attractor. To obtain a bi-stable system, a minimal connectivity level is needed in the network.
(PDF)

S1 Fig Effect of network connectivity *J* and noise amplitude *σ* on model (3) without AHP. A: Time-series and spectrograms of *h* (60s simulations) with SEF95 (blue curve) for *J* = 5.6 (upper), 6.6 (center) and 7.6 (lower). B: Mean value of the SEF95 for *J* ∈ [3.8, 10]. C: Time-series and spectrograms of *h* (60s simulations) with SEF95 (blue curve) for *σ* = 7 (upper), 11 (center) and 15 (lower). D: Mean value of the SEF95 for *σ* ∈ [5, 15]. Synaptic plasticity timescales: *τ* = 0.025s,*τ*_*r*_ = 0.5s and *τ*_*f*_ = 0.3s.
(TIF)

S2 Fig Effect of network connectivity *J* and noise amplitude *σ* on model (3) with AHP. A: Time-series and spectrograms of *h* (60s simulations) with SEF95 (blue curve) for *J* = 5.6 (upper), 6.6 (center) and 7.6 (lower). B: Mean value of the SEF95 in the upstates for *J* ∈ [3.8, 10]. C: Time-series and spectrograms of *h* (60s simulations) with SEF95 (blue curve) for *σ* = 7 (upper), 11 (center) and 15 (lower). D: Mean value of the SEF95 in the upstates for *σ* ∈ [5, 15]. Synaptic plasticity timescales: *τ* = 0.025s,*τ*_*r*_ = 0.5s and *τ*_*f*_ = 0.3s.
(TIF)

S3 Fig Effect of an input current *I*_*up*_ during the up states in model (3). A: Time-series and spectrograms of *h* (60s simulations, model (3) without AHP with *J* = 6.6, *σ* = 10, *τ* = 0.01s,*τ*_*r*_ = 0.2s and *τ*_*f*_ = 0.12s), with peak value of the oscillatory band, (black curve) for *I*_*up*_ = −20 (upper) 20 (center) and 80 (lower). B: Mean peak value of the oscillatory band for *I*_*up*_ ∈ [−80, 80]. C: Time-series and spectrograms of *h* (60s simulations, model (3) with AHP with *J* = 6.6, *σ* = 14, *τ* = 0.025s,*τ*_*r*_ = 0.5s and *τ*_*f*_ = 0.3*s*). D: Proportion of time spent in up vs down states for *I*_*up*_ = {−20, 20, 80} (*N* = 50 simulations of *T* = 5min, model (3) with AHP).
(TIF)

S4 Fig Contribution of the three components of model (6) for a constant input. A: Time-series of mean voltage *h*, spectrogram, facilitation *x* and depression *y* of system 3 (120s simulations) for the excitatory network with AHP (*U*/*D*, left: *τ* = 0.025s, *τ*_*f*_ = 0.3s,*τ*_*r*_ = 0.5s), the inhibitory network (*NR*, center) and the excitatory network without AHP (*α*, right: *τ* = 0.005s, *τ*_*f*_ = 0.12s, *τ*_*r*_ = 0.2s) with a constant input *I*_*i*_ = 50 on the inhibitory network (red line). B: Trajectories in the *h* − *x* − *y* phase space of each component (*U*/*D*, pink, left, NR black, center and *α*, green, right).
(TIF)

S5 Fig Contribution of the three components of model (6) for a step input. A: Time-series of mean voltage *h*, spectrogram, facilitation *x* and depression *y* of system 3 (120s simulations) for the excitatory network with AHP (*U*/*D*, left: *τ* = 0.005s, *τ*_*f*_ = 0.06s,*τ*_*r*_ = 0.12s), the inhibitory network (NR, center) and the excitatory network without AHP (*α*, right: *τ* = 0.005s, *τ*_*f*_ = 0.06s,*τ*_*r*_ = 0.12s) with a step input *I*_*i*_ = 1000 at 40–60s on the inhibitory network (red line). B: Trajectories in the *h* − *x* − *y* phase space of each component (*U*/*D*, pink, left, NR black, center and *α*, green, right).
(TIF)

S6 Fig Schematic of the fragmentation analysis using the spectrogram. (TIF)

S7 Fig Phase-space of system (3) without AHP. A: 3D phase-space of the system with the two attractors *A*_*Down*_ (purple, resp. *A*_*Up*_, red) and saddle-point *S* (cyan) with its 2-dimensional stable manifold Γ (blue surface) which defines the separatrix. Stable trajectories (black curves) and unstable manifold of *S* (grey) and deterministic trajectories starting below (purple, resp. above light red) Γ falling to *A*_*Down*_ (resp. *A*_*Up*_). Top view (A.1 upper), inset around *A*_*Down*_ and *S* (A.2), inset around *A*_*Up*_ where deterministic trajectories oscillate at their eigenfrequency *ω*_*Up*_ (light red, A.3), schematic summary of the entire phase-space (A.4). B: Stochastic trajectory lasting *T* = 30*s* with *σ* = 10 starting at *A*_*Down*_ and oscillating around *A*_*Up*_. C: (*h*, *x*, *y*)-time series of a stochastic trajectory, with the spectrogram of the mean voltage *h* and SEF95 (blue curve).
(TIF)

S8 Fig Phase-space of system (3) with AHP. A: 3D phase-space of the system with the two attractor points *A*_*Down*_ (purple), *A*_*Up*_ (red) and the saddle-point *S* (cyan) with its 2-dimensional stable manifold Γ (blue surface) which defines the separatrix. Stable trajectories (black curves) and unstable manifold of *S* (grey) and deterministic trajectories starting below (purple, resp. above light red) Γ falling to *A*_*Down*_ (resp. *A*_*Up*_). The phase-space is separated into 3 subspaces defining the different dynamics: fast Ω_*fast*_ (above pink and orange meshes), medium Ω_*mAHP*_ (below the orange mesh) and slow Ω_*sAHP*_ (below the pink mesh). Top view (A.1 upper), side view (A.1 lower), inset around *A*_*Down*_ and *S* (A.2), inset around *A*_*Up*_ (A.3), schematic summary of the entire phase-space (A.4). B: Stochastic trajectory lasting *T* = 30*s* with *σ* = 10 starting at *A*_*Down*_ and oscillating between *A*_*Up*_ and *A*_*Down*_. C: (*h*, *x*, *y*)-time series of a stochastic trajectory, with the spectrogram of the mean voltage *h* and SEF95 (blue curve).
(TIF)

S1 Appendix Supplementary methods. Detailed description of the phase-space of model (3) with and without AHP, numerical construction of the separatrix and time-series segmentation into Up and Down states.
(PDF)
