# Supplementary material for: Emergence and fragmentation of the alpha-band driven by neuronal network dynamics
Source: PLoS Comput Biol. 2021 Dec 6;17(12):e1009639. doi: 10.1371/journal.pcbi.1009639 (PMC8675921; doi:10.1371/journal.pcbi.1009639)
Supplement: S1 Table — For models (5) and (6), the inhibitory population is always without AHP and excitatory populations can be with or without AHP. For model (6) E1 corresponds to the network with AHP (U/D), and E2 to the network without AHP (α). The parameters values are chosen in agreement with [23, 24, 26, 29, 46]. The timescales τf and τr and the facilitation and depression rates K and L have been scaled in order to obtain oscillations in the range 5 − 15Hz around the Up state attractor AUp. The network connectivity values Jii (i = {I, E1, E2}) are adjusted (J = 4.21 in [29]) in order to reach a bifurcation that transforms the fixed point AUp from a saddle-point in [29] to an attractor. To obtain a bi-stable system, a minimal connectivity level is needed in the network. (PDF) [file pcbi.1009639.s001.pdf]

	1 population		2 populations		3 populations		
	no AHP	AHP	no AHP & I	AHP	same E	no AHP & I	AHP
τ	0.005 (α) - 0.01s (θ)	0.025s	0.005 (α) - 0.01s	0.025s	0.005s	0.005-0.07s	0.025s
τ_r	0.2 - 0.5s	0.5s	0.2 - 0.5s	0.5s	0.1s	0.1 -0.2s	0.5s
τ_f	0.12 - 0.3s	0.3s	0.12 - 0.3s	0.3s	0.06s	0.06 - 0.12s	0.3s
τ_{mAHP}		0.3s		0.12s	0.06s		0.12s
τ_{sAHP}		1s - 10.5s		1s	0.5s		1s
$J_{E_1E_1}$	5.6 - 8.6		6.8		5.6	6.5	
J_{E_1I}			5.1		5.6	6.5	
J_{IE_1}			3.4		4.48	16.25	
J_{II}			8.5		5.6	3.25	
$J_{E_1E_2}$					2.8	1.3	
$J_{E_2E_1}$					1.12	1.3	
J_{E_2I}					0	0	
J_{IE_2}					4.48	16.25	
$J_{E_2E_2}$					4.2	6.5	
σ	5 - 15		2.75 (σ_I) 5.5 (σ_E)		10 (σ_T) 3 ($\sigma_{C,R}$)	2.5 ($\sigma_{T,C,R}$)	
T_{AHP}		-30		-30	-30		-30
K	0.5 Hz						
L	0.3 Hz						
X	0.06						

Table 1: Models 3 (1 population), 5 (2 populations) and 6 (3 populations) parameters (see Main text, Methods). For models (5) and (6), the inhibitory population is always without AHP and excitatory populations can be with or without AHP. For model (6) E_1 corresponds to the network with AHP (U/D), and E_2 to the network without AHP (α).
