# Supplementary figures and images for: Emergence and fragmentation of the alpha-band driven by neuronal network dynamics
Source: PLoS Comput Biol. 2021 Dec 6;17(12):e1009639. doi: 10.1371/journal.pcbi.1009639 (PMC8675921; doi:10.1371/journal.pcbi.1009639)

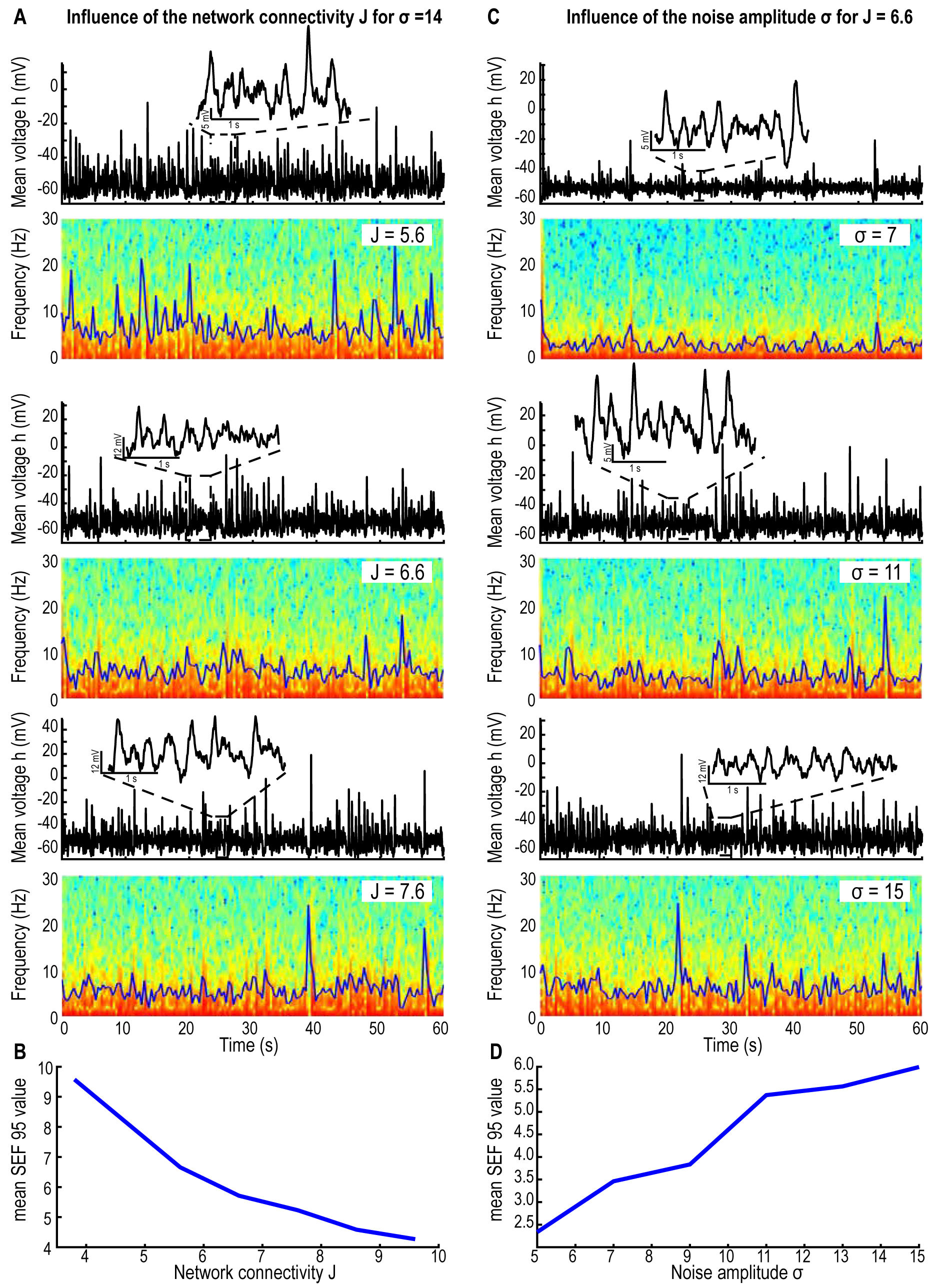

Supplement: S1 Fig — A: Time-series and spectrograms of h (60s simulations) with SEF95 (blue curve) for J = 5.6 (upper), 6.6 (center) and 7.6 (lower). B: Mean value of the SEF95 for J ∈ [3.8, 10]. C: Time-series and spectrograms of h (60s simulations) with SEF95 (blue curve) for σ = 7 (upper), 11 (center) and 15 (lower). D: Mean value of the SEF95 for σ ∈ [5, 15]. Synaptic plasticity timescales: τ = 0.025s,τr = 0.5s and τf = 0.3s. (TIF) [file pcbi.1009639.s002.tif]

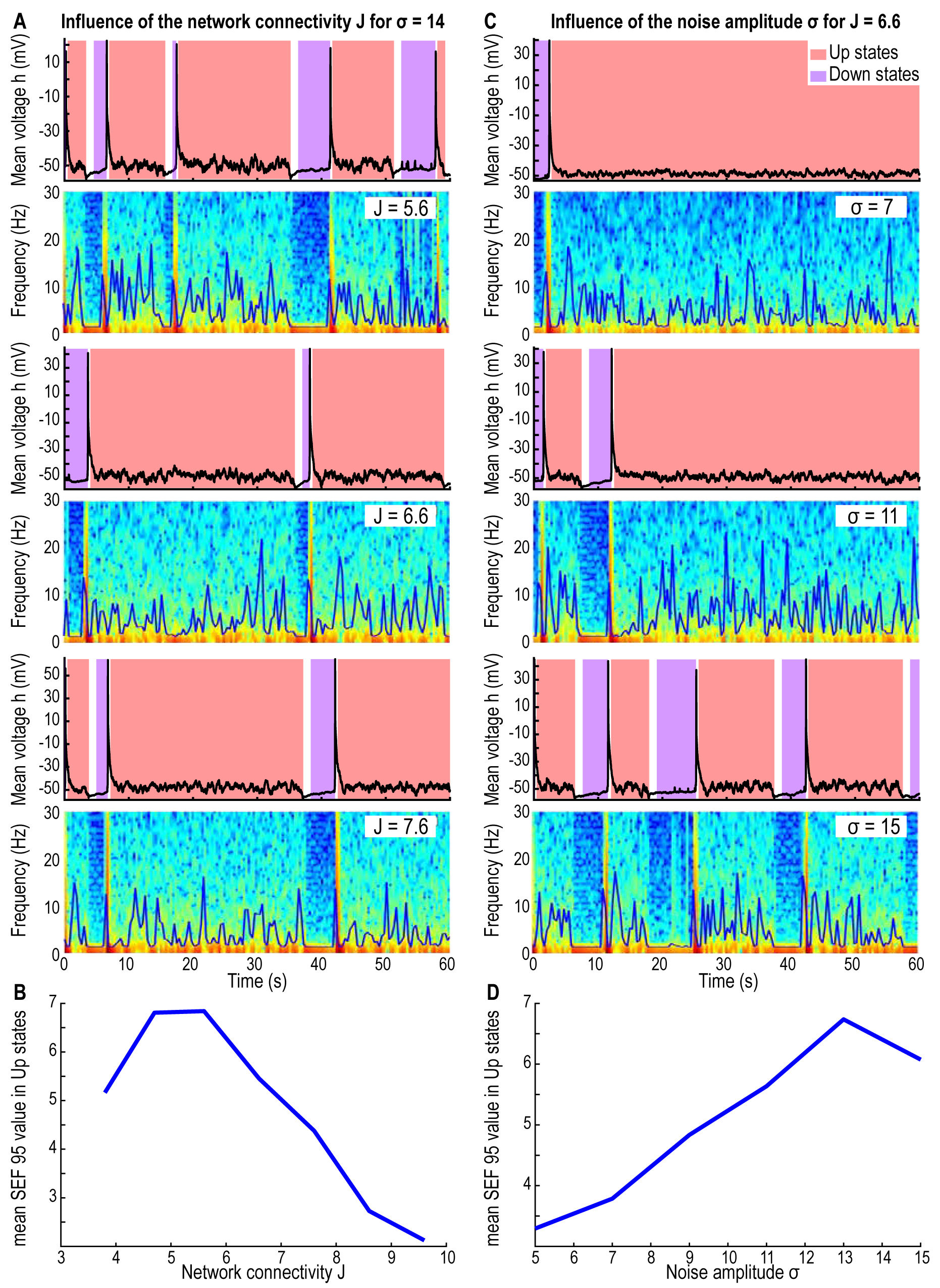

Supplement: S2 Fig — A: Time-series and spectrograms of h (60s simulations) with SEF95 (blue curve) for J = 5.6 (upper), 6.6 (center) and 7.6 (lower). B: Mean value of the SEF95 in the upstates for J ∈ [3.8, 10]. C: Time-series and spectrograms of h (60s simulations) with SEF95 (blue curve) for σ = 7 (upper), 11 (center) and 15 (lower). D: Mean value of the SEF95 in the upstates for σ ∈ [5, 15]. Synaptic plasticity timescales: τ = 0.025s,τr = 0.5s and τf = 0.3s. (TIF) [file pcbi.1009639.s003.tif]

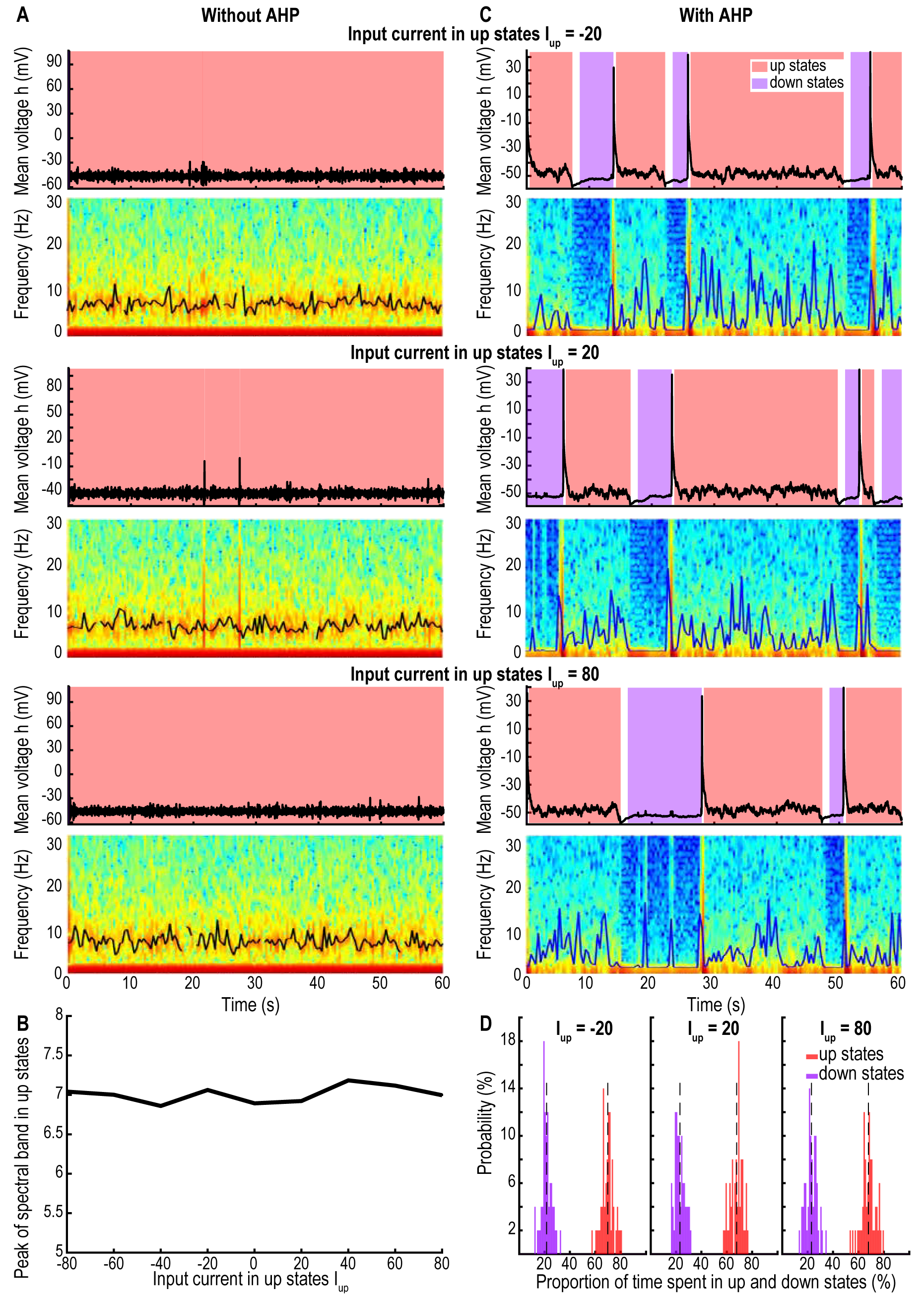

Supplement: S3 Fig — A: Time-series and spectrograms of h (60s simulations, model (3) without AHP with J = 6.6, σ = 10, τ = 0.01s,τr = 0.2s and τf = 0.12s), with peak value of the oscillatory band, (black curve) for Iup = −20 (upper) 20 (center) and 80 (lower). B: Mean peak value of the oscillatory band for Iup ∈ [−80, 80]. C: Time-series and spectrograms of h (60s simulations, model (3) with AHP with J = 6.6, σ = 14, τ = 0.025s,τr = 0.5s and τf = 0.3s). D: Proportion of time spent in up vs down states for Iup = {−20, 20, 80} (N = 50 simulations of T = 5min, model (3) with AHP). (TIF) [file pcbi.1009639.s004.tif]

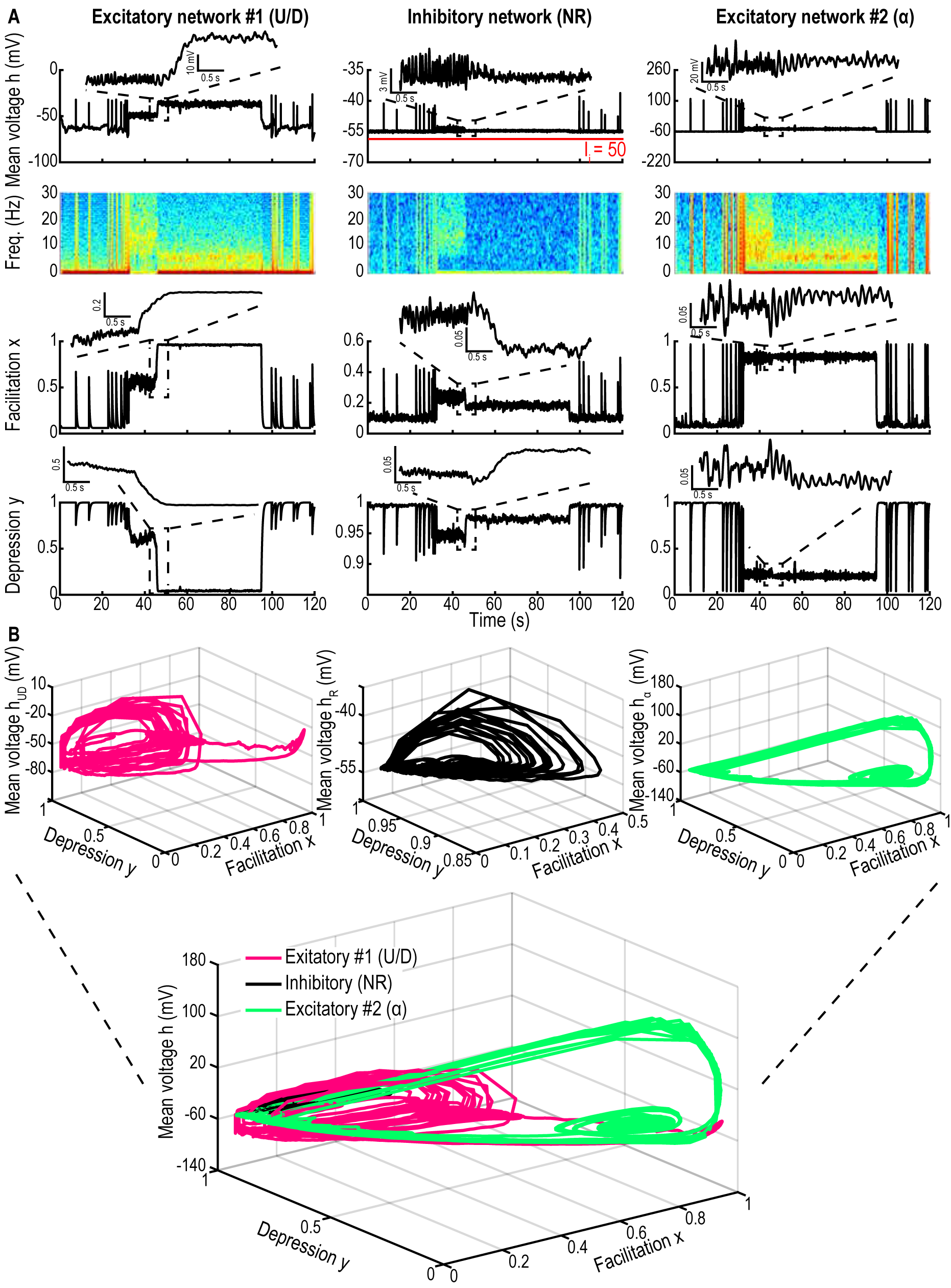

Supplement: S4 Fig — A: Time-series of mean voltage h, spectrogram, facilitation x and depression y of system 3 (120s simulations) for the excitatory network with AHP (U/D, left: τ = 0.025s, τf = 0.3s,τr = 0.5s), the inhibitory network (NR, center) and the excitatory network without AHP (α, right: τ = 0.005s, τf = 0.12s, τr = 0.2s) with a constant input Ii = 50 on the inhibitory network (red line). B: Trajectories in the h − x − y phase space of each component (U/D, pink, left, NR black, center and α, green, right). (TIF) [file pcbi.1009639.s005.tif]

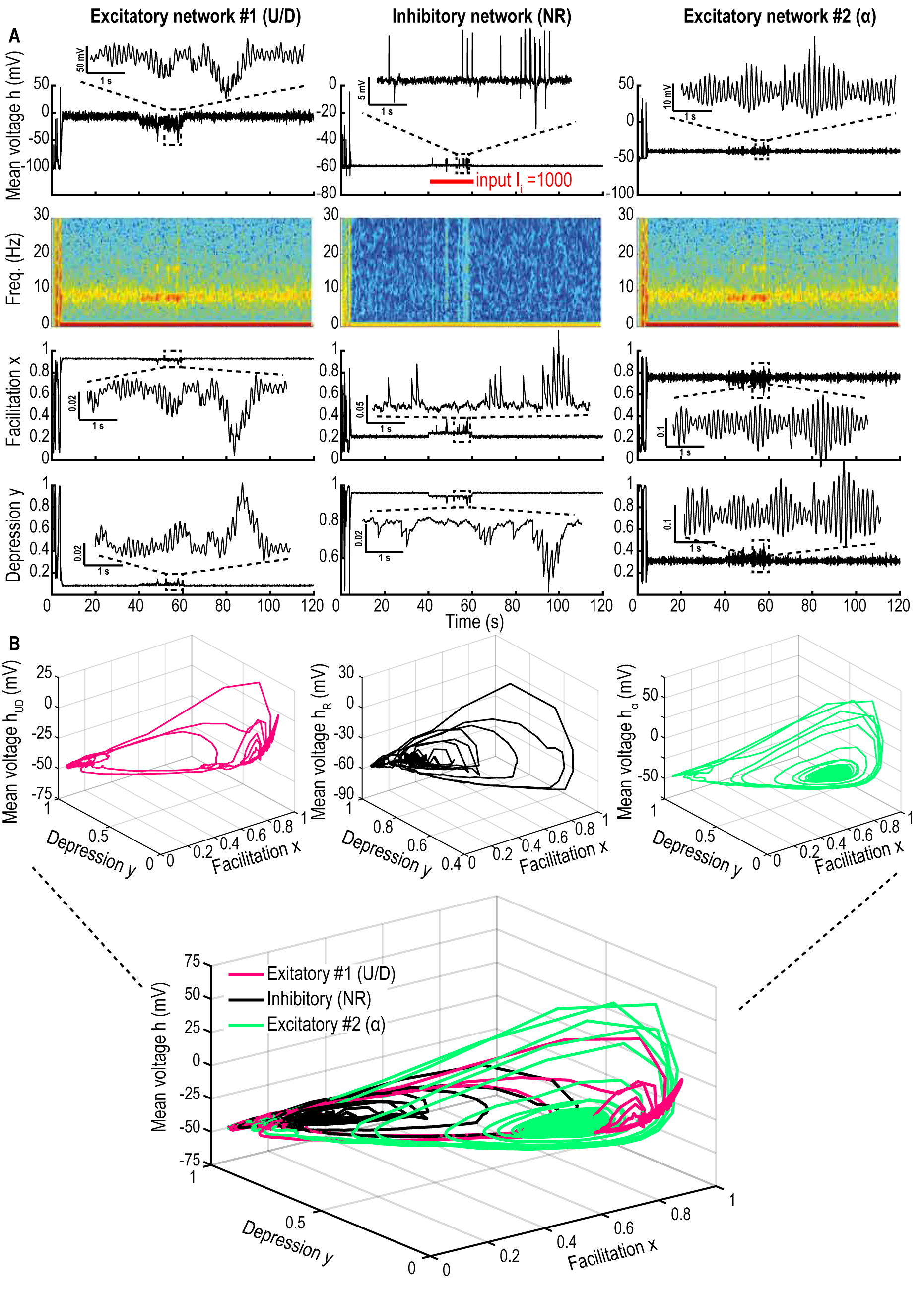

Supplement: S5 Fig — A: Time-series of mean voltage h, spectrogram, facilitation x and depression y of system 3 (120s simulations) for the excitatory network with AHP (U/D, left: τ = 0.005s, τf = 0.06s,τr = 0.12s), the inhibitory network (NR, center) and the excitatory network without AHP (α, right: τ = 0.005s, τf = 0.06s,τr = 0.12s) with a step input Ii = 1000 at 40–60s on the inhibitory network (red line). B: Trajectories in the h − x − y phase space of each component (U/D, pink, left, NR black, center and α, green, right). (TIF) [file pcbi.1009639.s006.tif]

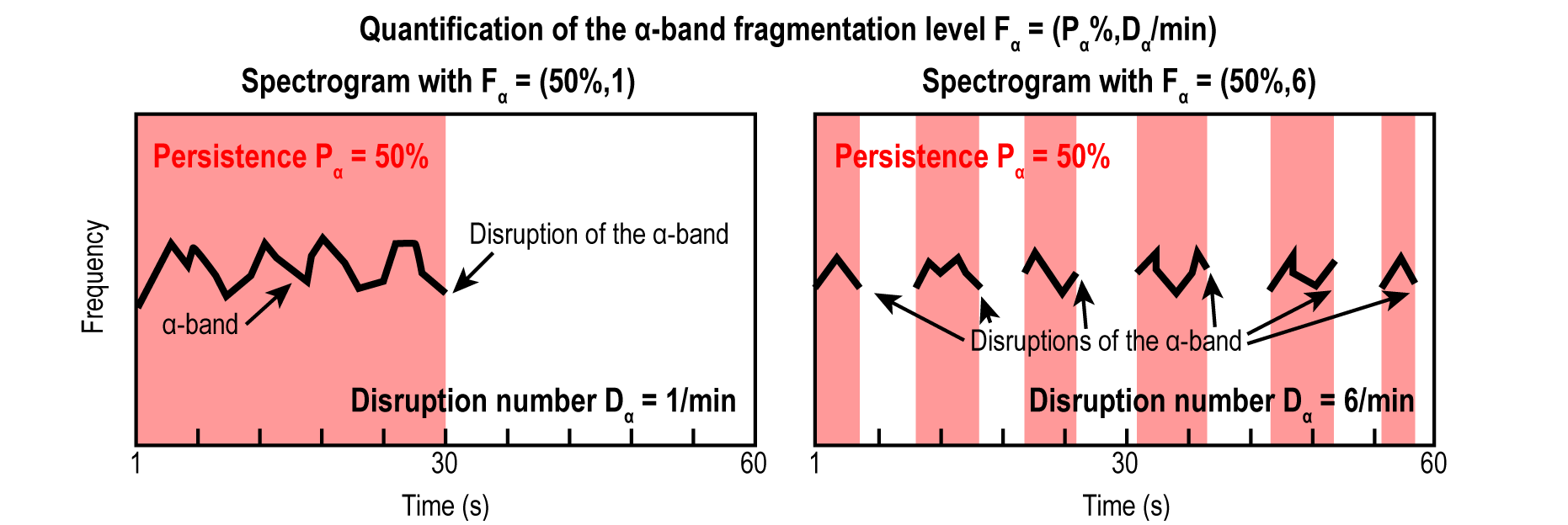

Supplement: S6 Fig — (TIF) [file pcbi.1009639.s007.tif]

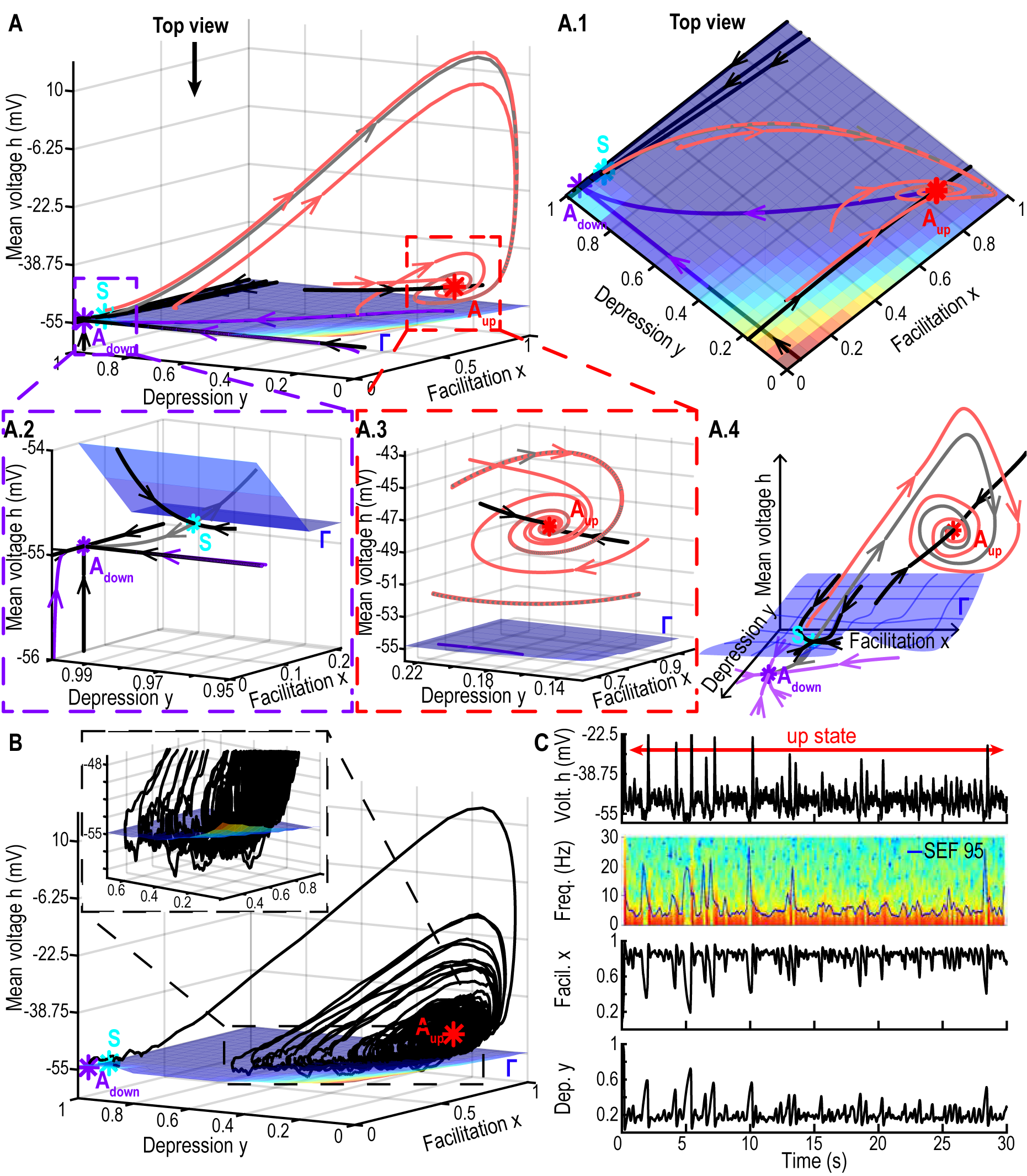

Supplement: S7 Fig — A: 3D phase-space of the system with the two attractors ADown (purple, resp. AUp, red) and saddle-point S (cyan) with its 2-dimensional stable manifold Γ (blue surface) which defines the separatrix. Stable trajectories (black curves) and unstable manifold of S (grey) and deterministic trajectories starting below (purple, resp. above light red) Γ falling to ADown (resp. AUp). Top view (A.1 upper), inset around ADown and S (A.2), inset around AUp where deterministic trajectories oscillate at their eigenfrequency ωUp (light red, A.3), schematic summary of the entire phase-space (A.4). B: Stochastic trajectory lasting T = 30s with σ = 10 starting at ADown and oscillating around AUp. C: (h, x, y)-time series of a stochastic trajectory, with the spectrogram of the mean voltage h and SEF95 (blue curve). (TIF) [file pcbi.1009639.s008.tif]

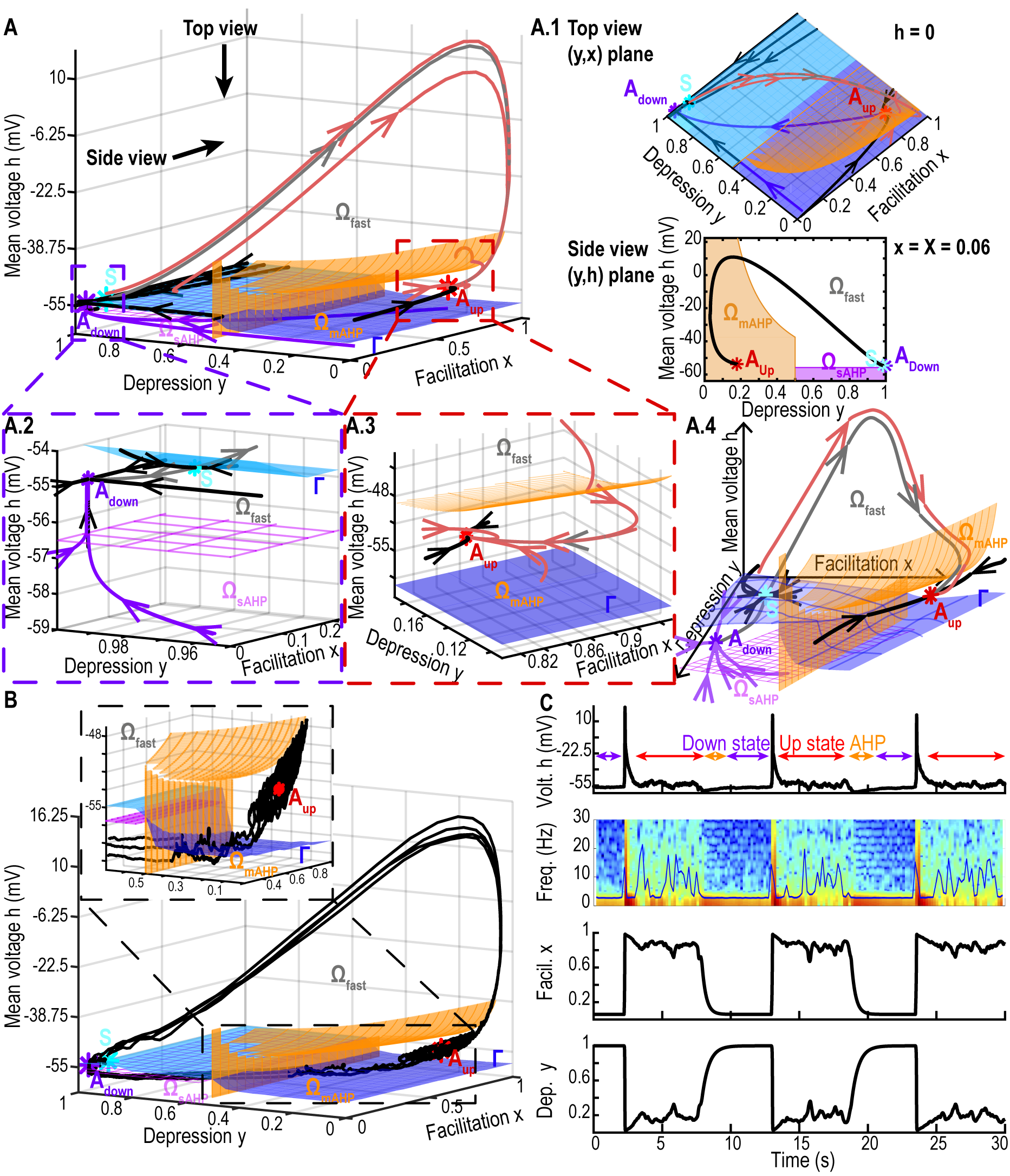

Supplement: S8 Fig — A: 3D phase-space of the system with the two attractor points ADown (purple), AUp (red) and the saddle-point S (cyan) with its 2-dimensional stable manifold Γ (blue surface) which defines the separatrix. Stable trajectories (black curves) and unstable manifold of S (grey) and deterministic trajectories starting below (purple, resp. above light red) Γ falling to ADown (resp. AUp). The phase-space is separated into 3 subspaces defining the different dynamics: fast Ωfast (above pink and orange meshes), medium ΩmAHP (below the orange mesh) and slow ΩsAHP (below the pink mesh). Top view (A.1 upper), side view (A.1 lower), inset around ADown and S (A.2), inset around AUp (A.3), schematic summary of the entire phase-space (A.4). B: Stochastic trajectory lasting T = 30s with σ = 10 starting at ADown and oscillating between AUp and ADown. C: (h, x, y)-time series of a stochastic trajectory, with the spectrogram of the mean voltage h and SEF95 (blue curve). (TIF) [file pcbi.1009639.s009.tif]
