# Supplementary material for: Emergence and fragmentation of the alpha-band driven by neuronal network dynamics
Source: PLoS Comput Biol. 2021 Dec 6;17(12):e1009639. doi: 10.1371/journal.pcbi.1009639 (PMC8675921; doi:10.1371/journal.pcbi.1009639)
Supplement: S1 Appendix — Detailed description of the phase-space of model (3) with and without AHP, numerical construction of the separatrix and time-series segmentation into Up and Down states. (PDF) [file pcbi.1009639.s010.pdf]

Mathematical analysis of the phase-space associated with the mean-field depression-facilitation model

We shall describe here the phase-space of the dynamical system (3) with and without AHP. In a first section we describe the three critical points (two attractors and a saddle-point) and the linearized dynamics around each point, in a second section we describe the numerical method used to obtain the shape of the separatrix delimiting the basins of attraction of each attractor and in the third section we describe the time-series segmentation into Up and Down states.

Description of the three critical points of the phase-space of network model (3)

The phase-space of the deterministic system (3) contains three critical points that we shall analyze now.

Down state attractor point A_{Down}

The basin of attraction of the critical point $A_{Down} = (0, X, 1)$ (S7 Fig A and S8 Fig A, purple) defines the Down state region. The Jacobian at this point is

$$J_{A_{Down}} = \begin{pmatrix} \dfrac{-1 + JX}{\tau} & 0 & 0 \\ K(1-X) & -\dfrac{1}{\tau_f} & 0 \\ LX & 0 & -\dfrac{1}{\tau_r} \end{pmatrix}. \tag{S1}$$

The eigenvalues are $\left(\lambda_1^{A_{Down}}, \lambda_2^{A_{Down}}, \lambda_3^{A_{Down}}\right) = \left(\dfrac{JX-1}{\tau}, -\dfrac{1}{\tau_f}, -\dfrac{1}{\tau_r}\right)$. When the connectivity J varies in the range $[5.6, 8.6]$, the attractor A_{Down} is a stable-node since the first eigenvalue is negative as long as $J \leq \dfrac{1}{X} \approx 16.67$. For $J \in [5.6, 8.6], \tau = 0.025s, \tau_f = 0.3s, \tau_r = 0.5s$ and the parameter values of Table S1, we obtain that $\lambda_1^{A_{Down}} \in [-19, -27]$, $\lambda_2^{A_{Down}} \approx -3.33$ and $\lambda_3^{A_{Down}} \approx -2$. The dynamics at this point is identical for the systems exhibiting AHP or not.

Up state attractor A_{Up}

The second critical point (S7 Fig A and S8 Fig A, red) is obtained by solving

$$\begin{aligned} x_{Up} &= \frac{\tau_f K(J+1) + LX\tau_r + \sqrt{\Delta}}{2(J\tau_f K + L\tau_r)} \\ y_{Up} &= \frac{1}{Jx_{Up}} \\ h_{Up} &= T + T_0 + \frac{x_{Up} - X}{\tau_f K(1 - x_{Up})}, \end{aligned} \tag{S2}$$

where

$$\Delta = (\tau_f K(J+1) + LX\tau_r)^2 - 4(J\tau_f K + L\tau_r)\tau_f K. \tag{S3}$$

The dynamics around this point depends on whether the system exhibits AHP or not, we will now describe these two cases.

1. **Neuronal network without AHP:** For that system, the resting membrane potential T_0 and the recovery timescale τ_0 of the mean voltage h are constant in the entire phase-space. The numerical range of values for the position of the critical point A_{Up} for $J \in [5.6, 8.6], \tau = 0.01s, \tau_f = 0.2s, \tau_r = 0.12s$ and parameters values from Table S1 is $A_{Up} = (h_{A_{Up}} \in [73.15, 124.59]$, $x_{A_{Up}} \in [0.83, 0.89]$, $y_{A_{Up}} \in [0.22, 0.13])$. The Jacobian at this point is

$$J_{A_{Up}} = \begin{pmatrix} 0 & \dfrac{Jy_{Up}(h_{Up} - T - T_0)^+}{\tau_0} & \dfrac{Jx_{Up}(h_{Up} - T - T_0)^+}{\tau_0} \\ K(1 - x_{Up}) & -\dfrac{1}{\tau_f} - K(h_{Up} - T - T_0)^+ & 0 \\ -\dfrac{L}{J} & -Ly_{1,2}(h_{Up} - T - T_0)^+ & -\dfrac{1}{\tau_r} - Lx_{Up}(h_{Up} - T - T_0)^+. \end{pmatrix} \tag{S4}$$

   With the present parameters, $J_{A_{Up}}$ has one real negative and two complex conjugate eigenvalues with negative real part thus A_{Up} is a stable-focus: $\lambda_1^{A_{Up}} \in [-55.71, -79.40]$ for the real eigenvalue and the two complex conjugate eigenvalues are

$$\lambda_{2,3}^{A_{Up}} \in [-6.16, -14.73] \pm i[36.78, 51.87].$$

2. **Neuronal network exhibiting AHP:** the Up state attractor A_{Up} is situated in the subspace of medium dynamics with hyperpolarization Ω_{mAHP} (S8 Fig A-B, orange) where $T_0 = T_{AHP} = -30$ and $\tau_0 = \tau_{m,AHP} \in [0.06, 0.3]$s. For $J \in [5.6, 8.6], \tau = 0.025s, \tau_f = 0.3s, \tau_r = 0.5s$ the position of A_{Up} is now $A_{Up} = \left(h_{A_{Up}} \in [-0.74, 19.84], x_{A_{Up}} \in [0.83, 0.89], y_{A_{Up}} \in [0.22, 0.13]\right)$. Here, the eigenvalues of $J_{A_{Up}}$ are real and negative, thus for the system with AHP A_{Up} is a stable-node. The numerical values are now $\lambda_1^{A_{Up}} \in [-34.01, -43.96]$, $\lambda_2^{A_{Up}} \in [-11.67, -18.94]$ and $\lambda_3^{A_{Up}} \in [-3.96, -3.65]$.

Saddle-point S

The third critical point S (S7 Fig A and S8 Fig A, cyan) is solution of equations

$$\begin{aligned} x_S &= \frac{\tau_f K(J+1) + LX\tau_r - \sqrt{\Delta}}{2(J\tau_f K + L\tau_r)} \\ y_S &= \frac{1}{Jx_S} \\ h_S &= T + T_0 + \frac{x_S - X}{\tau_f K(1 - x_S)}, \end{aligned} \tag{S5}$$

for $J \in [5.6, 8.6], \tau = 0.01s, \tau_f = 0.2s, \tau_r = 0.12s$ and the parameters are presented in Table S1, we get $A_S = (h_S \in [2.52, 1.08], x_S \in [0.18, 0.12], y_S \in [0.97, 0.99])$. The Jacobian at S does not

depend on whether the system exhibits AHP or not and it has one real positive and two real negative eigenvalues, it is thus a saddle-node with an unstable manifold of dimension one and a stable manifold of dimension two. With the present parameters, we obtain $\lambda_1^S \in [-28.80, -25.03]$, $\lambda_2^S \in [18.96, 16.08]$ and $\lambda_3^S \in [-4.89, -4.97]$. Finally, the stable two-dimensional manifold Γ defines the separatrix between the basins on attraction of Down A_{Down} and Up A_{Up} states.

Numerical construction of the separatrix

To represent the stable manifold Γ of the saddle-point S, we use the following algorithm based on numerical approximations (S7 Fig A-B and S8 Fig A-B, blue surface). Since Γ defines the separatrix between the two basins of attraction for the attractors A_{Down} and A_{Up}, we ran simulations of the noiseless dynamics for $\sigma = 0$ of system (1) with the initial condition sampling the entire phase space. We used grid points $(h_i, x_i, y_i) \in [-35, 500] \times [0, 1], \times[0, 1])$ with $\delta_h = 1, \delta_x = \delta_y = 0.05$. Each initial point was then attributed to the basin of attraction of the attractor at which the corresponding trajectory ended. The separatrix Γ is defined as the border between the set of initial points falling into the basin of A_{Down} and those falling into the basin of A_{Up}.
This separatrix does not define a bounded domain for neither attractor but rather separates the entire phase-space in two subdomains, one above Γ leading to the Up state and the other one below Γ to the Down state .

Segmentation of the time-series to detect Up and Down states

To determine whether the neuronal population is in an Up or a Down state, we segmented the simulated time-series according to the following criteria:

- the Up states are defined in the subspace $\{x \geq x_{Up} = 0.5 \& h \leq h_{Up} = 0.175 h_{max}\}$,
- the Down states are defined when $\{y \geq y_{Down} = 0.95\}$

We added the threshold on h for the Up state detection because we do not want to count the bursts, defining the transition from Down to Up, as an Up state.
To determine the proportion of time spent in Up vs Down state for one neuronal population with AHP (Fig 3C-D, main text), we ran simulations of system (3) with AHP for $N = 100$ trajectories of duration $T = 600s$ with $J \in \{5.6, 6.6, 7.6\}$ and $\sigma = 14$.
Similarly, for the model (5) with two populations (Fig 4B-D, main text), we segmented the time-series of the excitatory population for $N = 100$ trajectories of duration $T = 600s$.
Finally for the three population network (6), we segmented the time-series of the excitatory network α without AHP ($N = 100$ trajectories of duration $T = 300s$).

Numerical methods

All simulations were run in Matlab, using Runge-Kutta 4 scheme with a time step $\Delta t = 0.005$s. We also tried $\Delta t = 0.001$s and obtained the same results, thus ensuring stability.
